# Supplementary material for: Modeling Emergency Department crowding: Restoring the balance between demand for and supply of emergency medicine
Source: PLoS One. 2021 Jan 12;16(1):e0244097. doi: 10.1371/journal.pone.0244097 (PMC7802975; doi:10.1371/journal.pone.0244097)
Supplement: S1 Table — (DOCX) [file pone.0244097.s001.docx]

**S1 Table. Summary table of input parameters**

| **Parameter** | **Value** | **Unit** | **Source** |
| --- | --- | --- | --- |
| ***Registration and Triage*** |  |  |  |
| Arrival | Time series | Patient/minute | Patient records |
| Average registration time | [3,5,7] | Minute | Observational study |
| Average triage time | [4,5,7] | Minute | Observational study |
| Distribution to care pathway | Time series | Dimensionless | Patient records |
|  | | | |
| ***Critical care area (CCA)*** |  |  |  |
| Doctor per patient | 1 | Doctor/patient | Expert opinion |
| Time to adjust | 1 | Minute | Expert opinion |
| Number of doctors | Time series | Doctor | Observational study |
| Consult time | [10,15,20] | Minute | Observational study |
| Patient per doctor | 1 | Patient/doctor | Expert opinion |
| New arrival | Time series | Patient/minute | Patient records |
| Fraction to observation | Time series | Dimensionless | Observational study |
| Fraction admission | 0.1 | Dimensionless/minute | Observational study |
| Average waiting time (observation) | [30,60,90] | Minutes | Observational study |
| Lab and investigation time | [35,45,60] | Minute | Observational study |
| Fraction to home | 0.12 | Dimensionless | Observational study |
| Distribution to observation ward |  |  |  |
| P1 | 0.08 | Dimensionless | Observational study |
| P2 | 0.11 | Dimensionless | Observational study |
| P3 | 0 | Dimensionless | Observational study |
| P4 | 0 | Dimensionless | Observational study |
|  | | | |
| ***Ambulatory care (AMB)*** |  |  |  |
| Doctor per patient | 1 | Doctor/patient | Expert opinion |
| Time to adjust | 1 | Minute | Expert opinion |
| Number of doctors | Time series | Doctor | Observational study |
| Consult time | [10,15.5,20] | Minute | Observational study |
| Patient per doctor | 1 | Patient/doctor | Expert opinion |
| Distribution to observation ward |  |  |  |
| P1 | 0.08 | Dimensionless | Observational study |
| P2 | 0.05 | Dimensionless | Observational study |
| P3 | 0.1 | Dimensionless | Observational study |
| P4 | 0 | Dimensionless | Observational study |
| Fraction admission | 0.1 | Dimensionless/minute | Observational study |
| Average waiting time (observation) | [30,60,90] | Minute | Observational study |
|  | | | |
| ***Isolation care (ISO)*** |  |  |  |
| Doctor per patient | 1 | Doctor/patient | Expert opinion |
| Time to adjust | 1 | Minute | Expert opinion |
| Number of doctors | 1 | Doctor | Observational study |
| Consult time | [20,30,45] | Minute | Observational study |
| Patient per doctor | 1 | Patient/doctor | Expert opinion |
| Distribution to observation | Time series | Dimensionless | Observational study |
| Fraction admitted isolation | 0.1 | Dimensionless | Observational study |
| Average waiting time (observation) | ]30.60.90] | Minute | Observational study |
| Pharmacy and payment waiting time | [10,15,30] | minute | Observational study |
|  | | | |
| ***Observation ward/discharge (OW)*** |  |  |  |
| Observation capacity | 45 | Bed | Expert opinion |
| Patient per bed | 1 | Patient/bed | Expert opinion |
| Time to make bed available | [10,15,25] | Minute | Expert opinion |
| Average time to admit patients | [100,120,150] | Minute | Observational study |
| Fraction of patient to pharm/payment |  |  |  |
| P1 | 0.156 | Dimensionless | Observational study |
| P2 | 0.156 | Dimensionless | Observational study |
| P3 | 0.156 | Dimensionless | Observational study |
| P4 | 0 | Dimensionless | Observational study |
| Pharmacy and payment waiting time | [10,15,30] | Minute | Observational study |
