# Supplementary material for: Modeling Emergency Department crowding: Restoring the balance between demand for and supply of emergency medicine
Source: PLoS One. 2021 Jan 12;16(1):e0244097. doi: 10.1371/journal.pone.0244097 (PMC7802975; doi:10.1371/journal.pone.0244097)
Supplement: S2 Table — (DOCX) [file pone.0244097.s002.docx]

**S2 Table. Variable list with full names and abbreviations.**

| **Variable name** | **Abbreviation** |
| --- | --- |
| ***Registration and triage*** |  |
| Patient waiting for registration | $P\left( t \right)$ |
| New patient arrivals | $a\left( t \right)$ |
| Patients moving from registration to triage | $g\left( t \right)$ |
| Patients waiting for registration at time zero | $P(t_{0})$ |
| Average registration time | $RT$ |
| Time | $\left( t \right)$ |
| Patients waiting for triage | $B\left( t \right)$ |
| Patients triage into treatment priority—P1, P2, P3, P4 | $\left( j \right)$ |
| Patient triage to critical care by treatment priority | ${cca}_{j}\left( t \right)$ |
| Patients triage to ambulatory care by treatment priority | ${ab}_{j}\left( t \right)$ |
| Patients triage to isolation care by treatment priority | ${is}_{j}(t)$ |
| Patients waiting for triage at time zero | $B(t_{0})$ |
| Fraction of patients triaged to critical care by treatment priority | ${fcca}_{j}(t)$ |
| Fraction of patients triaged to ambulatory care by treatment priority | ${fab}_{j}(t)$ |
| Fraction of patients triaged to isolation care by treatment priority | ${fis}_{j}(t)$ |
| Delta of time | $dt$ |
|  |  |
| ***Critical care pathways*** |  |
| Patients waiting for consultation by priority treatment | $C_{j}\left( t \right)$ |
| New ambulance arrivals by priority treatment | ${nab}_{j}\left( t \right)$ |
| Patient starting consultation by priority treatment | ${cs}_{j}(t)$ |
| Patients waiting for consultation by priority treatment at time zero | $C_{j}(t_{0})$ |
| ED doctors initiate consultation by priority treatment | ${nc}_{j}\left( t \right)$ |
| Patient per doctor ratio | $ppd$ |
| ED doctors consulting | $PCC\left( t \right)$ |
| Completion of consultation by priority treatment | ${cc}_{j}(t)$ |
| ED doctors consulting at time zero | $PCC(t_{0})$ |
| ED doctors initiate consultation for P1 patients | ${nc}_{p1}\left( t \right)$ |
| ED doctors initiate consultation for P2 patients | ${nc}_{p2}\left( t \right)$ |
| Minimum | $MIN$ |
| ED doctors available to initiate consultation | $pa\left( t \right)$ |
| P1 patients waiting for consultation | $C_{p1}\left( t \right)$ |
| P2 patients waiting for consultation | $C_{p2}\left( t \right)$ |
| Adjustment time | $AT$ |
| Doctor per patient ratio in the critical care area | $dpp$ |
| Consultation time | $CT$ |
| Maximum | $MAX$ |
| ED doctors allocated to critical care | $NP\left( t \right)$ |
| ED doctors consulting by patient priority | ${PCC}_{j}(t)$ |
| Summation | $\sum$ |
| Patients in consultation by patient priority | ${EP}_{j}\left( t \right)$ |
| Patients transferred to observation ward after consultation | ${co}_{j}\left( t \right)$ |
| Patient transferred for laboratory and investigation at consultation | ${cl}_{j}\left( t \right)$ |
| Patient discharged home after consultation | ${ch}_{j}(t)$ |
| Patients in consultation by patient priority at time zero | ${EP}_{j}\left( t_{0} \right)$ |
| P1 Patients transferred to observation ward after consultation | ${co}_{p1}\left( t \right)$ |
| P2 Patients transferred to observation ward after consultation | ${co}_{p2}\left( t \right)$ |
| Available beds in the observation ward | $avb\left( t \right)$ |
| P1 patients requiring referral to observation ward | ${cpo}_{p1}(t)$ |
| P2 patients requiring referral to observation ward | ${cpo}_{p2}(t))$ |
| Fraction of patients who require observation | $fb$ |
| Fraction of patients discharged home after consultation | $fh$ |
| Patients waiting for lab and investigation by priority treatment | ${PLI}_{J}\left( t \right)$ |
| Patient transferred to the observation ward by treatment priority | ${lo}_{j}\left( t \right)$ |
| Patient transferred to the lab and investigation by treatment priority | ${ld}_{j}\left( t \right)$ |
| Patients observed at waiting area by treatment priority | ${lw}_{j}(t)$ |
| Patients waiting for lab and investigation by priority treatment at time zero | ${PLI}_{j}\left( t_{0} \right)$ |
| Patient under observation in waiting area by treatment priority | ${POW}_{j}\left( t \right)$ |
| Patient discharged from waiting area by priority treatment | ${dw}_{j}\left( t \right)$ |
| Patient admitted to hospital from waiting area by priority treatment | ${cad}_{j}\left( t \right)$ |
| Patient under observation in waiting area by treatment priority at time zero | ${POW}_{j}\left( t_{0} \right)$ |
| P1 patient transferred to the observation ward | ${lo}_{p1}\left( t \right)$ |
| P2 patient transferred to the observation ward | ${lo}_{p2}\left( t \right)$ |
| P1 patients who have completed lab and investigation | ${al}_{p1}\left( t \right)$ |
| P2 patients who have completed lab and investigation | ${al}_{p2}\left( t \right)$ |
| Fraction of P1 patients who need observation ward after lab and investigation | ${fob}_{p1}$ |
| Fraction of P2 patients who need observation ward after lab and investigation | ${fob}_{p2}$ |
| Patients who have completed lab and investigation by treatment priority | ${al}_{j}\left( t \right)$ |
| P1 patients observed at waiting area | ${lw}_{p1}\left( t \right)$ |
| P2 patients observed at waiting area | ${lw}_{p2}\left( t \right)$ |
| Average waiting time for lab and investigation | $LIT$ |
| Average observation time | $wt$ |
| Fraction of patients waiting in the waiting area admitted | $fdd$ |
|  |  |
| ***Ambulatory care pathways*** |  |
| Patients waiting for consultation by patient priority | ${CAB}_{j}\left( t \right)$ |
| Consultation starts by patient priority | ${csAB}_{j}(t)$ |
| Patients waiting for consultation by patient priority at time zero | ${CAB}_{j}(t_{0})$ |
| ED physician consulting | $PCAB\left( t \right)$ |
| ED doctor become available and starts consultation by patient priority | ${ncAB}_{j}\left( t \right)$ |
| Completed consultation by patient priority | ${ccAB}_{j}(t)$ |
| ED physician consulting at time zero | $PCAB(t_{0})$ |
| Doctor patient ratio | $ppd$ |
| ED doctor available to starts consultation on P1 patients | ${ncAB}_{p1}\left( t \right)$ |
| ED doctor available to starts consultation on P2 patients | ${ncAB}_{p2}\left( t \right)$ |
| ED doctor available to starts consultation on P3 patients | ${ncAB}_{p3}\left( t \right)$ |
| ED doctor available to starts consultation on P4 patients | ${ncAB}_{p4}\left( t \right)$ |
| Available ED doctors to initiate consultation | $paAB\left( t \right)$ |
| P1 patients waiting for consultation | ${CAB}_{p1}\left( t \right)$ |
| P2 patients waiting for consultation | ${CAB}_{p2}\left( t \right)$ |
| P3 patients waiting for consultation | ${CAB}_{p3}\left( t \right)$ |
| P4 patients waiting for consultation | ${CAB}_{p4}\left( t \right)$ |
| ED physicians allocated to ambulatory care | $NPAB\left( t \right)$ |
| Patients in consultation by patient priority | ${EPAB}_{j}\left( t \right)$ |
| Patient referred for lab and investigation by patient priority | ${clAB}_{j}(t)$ |
| Patients in consultation by patient priority at time zero | ${EPAB}_{j}\left( t_{0} \right)$ |
| Patients waiting for lab and investigation by patient priority | ${PHIAB}_{j}\left( t \right)$ |
| Patients referred to observation ward by patient priority | ${ao}_{j}\left( t \right)$ |
| Patient discharged to home by patient priority | ${lwAB}_{j}\left( t \right)$ |
| Patients transferred to observation ward from waiting area by patient priority | ${ldAB}_{j}\left( t \right)$ |
| Patients waiting for lab and investigation by patient priority at time zero | ${PHIAB}_{j}\left( t_{0} \right)$ |
| Patient under observation in waiting area by patient priority | ${POWAB}_{j}\left( t \right)$ |
| Patients discharged from waiting area by patient priority | ${dwAB}_{j}\left( t \right)$ |
| Patients admitted from waiting area by patient priority | ${aAB}_{j}\left( t \right)$ |
| Patient under observation in waiting area by patient priority at time zero | ${POWAB}_{j}\left( t_{0} \right)$ |
| P1 patient discharged to home | ${lwAB}_{p1}\left( t \right)$ |
| P2 patient discharged to home | ${lwAB}_{p2}\left( t \right)$ |
| P3 patient discharged to home | ${lwAB}_{p3}\left( t \right)$ |
| P4 patient discharged to home | ${lwAB}_{p4}\left( t \right)$ |
| Patients who have finished lab and investigation by patient priority | ${ala}_{j}\left( t \right)$ |
| P1 patients who have finished lab and investigation | ${ala}_{p1}\left( t \right)$ |
| P2 patients who have finished lab and investigation | ${ala}_{p2}\left( t \right)$ |
| P3 patients who have finished lab and investigation | ${ala}_{p3}\left( t \right)$ |
| P4 patients who have finished lab and investigation | ${ala}_{p4}\left( t \right)$ |
| P1 patients referred to observation ward | ${ao}_{p1}\left( t \right)$ |
| P2 patients referred to observation ward | ${ao}_{p2}\left( t \right)$ |
| P3 patients referred to observation ward | ${ao}_{p3}\left( t \right)$ |
| P4 patients referred to observation ward | ${ao}_{p4}\left( t \right)$ |
| Fraction of patients who have finished lab and investigation and admitted to observation ward by patient priority | ${foba}_{j}$ |
| Fraction of P1 patients who have finished lab and investigation and admitted to observation ward | ${foba}_{p1}$ |
| Fraction of P2 patients who have finished lab and investigation and admitted to observation ward | ${foba}_{p2}$ |
| Fraction of P3 patients who have finished lab and investigation and admitted to observation ward | ${foba}_{p3}$ |
| Fraction of P4 patients who have finished lab and investigation and admitted to observation ward | ${foba}_{p4}$ |
| Fraction of patients observed in the waiting area admitted | $famb$ |
|  |  |
| ***Observation ward and discharge*** |  |
| Patients in observation ward by patient priority | ${OW}_{j}\left( t \right)$ |
| Patient admitted into the hospital from observation ward by patient priority | ${ah}_{j}\left( t \right)$ |
| Patients discharged home by patient priority | ${do}_{j}(t)$ |
| Patients in observation ward by patient priority at time zero | ${OW}_{J}\left( t_{0} \right)$ |
| Patients in pharmacy and payment by patient priority | ${PHAB}_{j}\left( t \right)$ |
| Patients leaving pharmacy and payment to home by patient priority | ${hab}_{j}(t)$ |
| Patients in pharmacy and payment by patient priority at time zero | ${PHAB}_{j}\left( t_{0} \right)$ |
| Patient leaving observation ward by patient priority | ${aobw}_{j}\left( t \right)$ |
| Average observation ward time | $ot$ |
| Fraction of patients discharged from observation ward | $df$ |
| Observation ward bed capacity | $bc\left( t \right)$ |
| Patient per bed ratio | $ppb$ |
| Time to make bed available | $ttba$ |
|  |  |
| ***Isolation care pathways*** |  |
| Patient waiting for consultation by patient priority | ${CIS}_{j}\left( t \right)$ |
| Patients starting consultation by patient priority | ${csIS}_{j}(t)$ |
| Patient waiting for consultation by patient priority at time zero | ${CIS}_{j}(t_{0})$ |
| ED doctors consulting | $PCIS\left( t \right)$ |
| ED doctors start consulting by patient priority | ${ncIS}_{j}\left( t \right)$ |
| Consultation is completed by patient priority | ${ccIS}_{j}\left( t \right)$ |
| ED doctors consulting at time zero | $PCIS(t_{0})$ |
| P1 patients consulting ED doctors | ${ncIS}_{p1}\left( t \right)$ |
| P2 patients consulting ED doctors | ${ncIS}_{p2}\left( t \right)$ |
| P3 patients consulting ED doctors | ${ncIS}_{p3}\left( t \right)$ |
| P4 patients consulting ED doctors | ${ncIS}_{p4}\left( t \right)$ |
| Available ED doctors to initiate consultation | $paIS\left( t \right)$ |
| P1 patient waiting for consultation | ${CIS}_{p1}\left( t \right)$ |
| P2 patient waiting for consultation | ${CIS}_{p2}\left( t \right)$ |
| P3 patient waiting for consultation | ${CIS}_{p3}\left( t \right)$ |
| P4 patient waiting for consultation | ${CIS}_{p4}\left( t \right)$ |
| ED doctors allocated to isolation area | $NPIS\left( t \right)$ |
| Patients in consultation by patient priority | ${EPIS}_{j}\left( t \right)$ |
| Patient transferred to observation ward by patient priority | ${coIS}_{j}\left( t \right)$ |
| Patients discharged after consultation by patient priority | ${cpIS}_{j}(t)$ |
| Patients in consultation by patient priority at time zero | ${EPIS}_{j}\left( t_{0} \right)$ |
| Patients in the observation ward by patient priority | ${OWIS}_{j}\left( t \right)$ |
| Patient discharged from the observation ward by patient priority | ${oph}_{j}\left( t \right)$ |
| Patients admitted from the observation ward by patient priority | ${ahis}_{j}(t)$ |
| Patients in the observation ward by patient priority at time zero | ${OWIS}_{j}\left( t_{0} \right)$ |
| Patients in pharmacy and payment by patient priority | ${PHIS}_{j}\left( t \right)$ |
| Patient leaving pharmacy and payment to home by patient priority | ${his}_{j}(t)$ |
| Patients in pharmacy and payment by patient priority at time zero | ${PHIS}_{j}\left( t_{0} \right)$ |
| Fraction of patients referred to observation ward | $fis$ |
| Patients leaving observation ward for discharge or admission | ${owd}_{j}\left( t \right)*$ |
| Fraction of patients admitted to the hospital | $fah$ |
| Average observation time | $otis$ |
| Average time at the pharmacy and payment | $ppt$ |
