# Supplementary material for: Modeling Emergency Department crowding: Restoring the balance between demand for and supply of emergency medicine
Source: PLoS One. 2021 Jan 12;16(1):e0244097. doi: 10.1371/journal.pone.0244097 (PMC7802975; doi:10.1371/journal.pone.0244097)
Supplement: S1 Fig — (DOCX) [file pone.0244097.s003.docx]

| 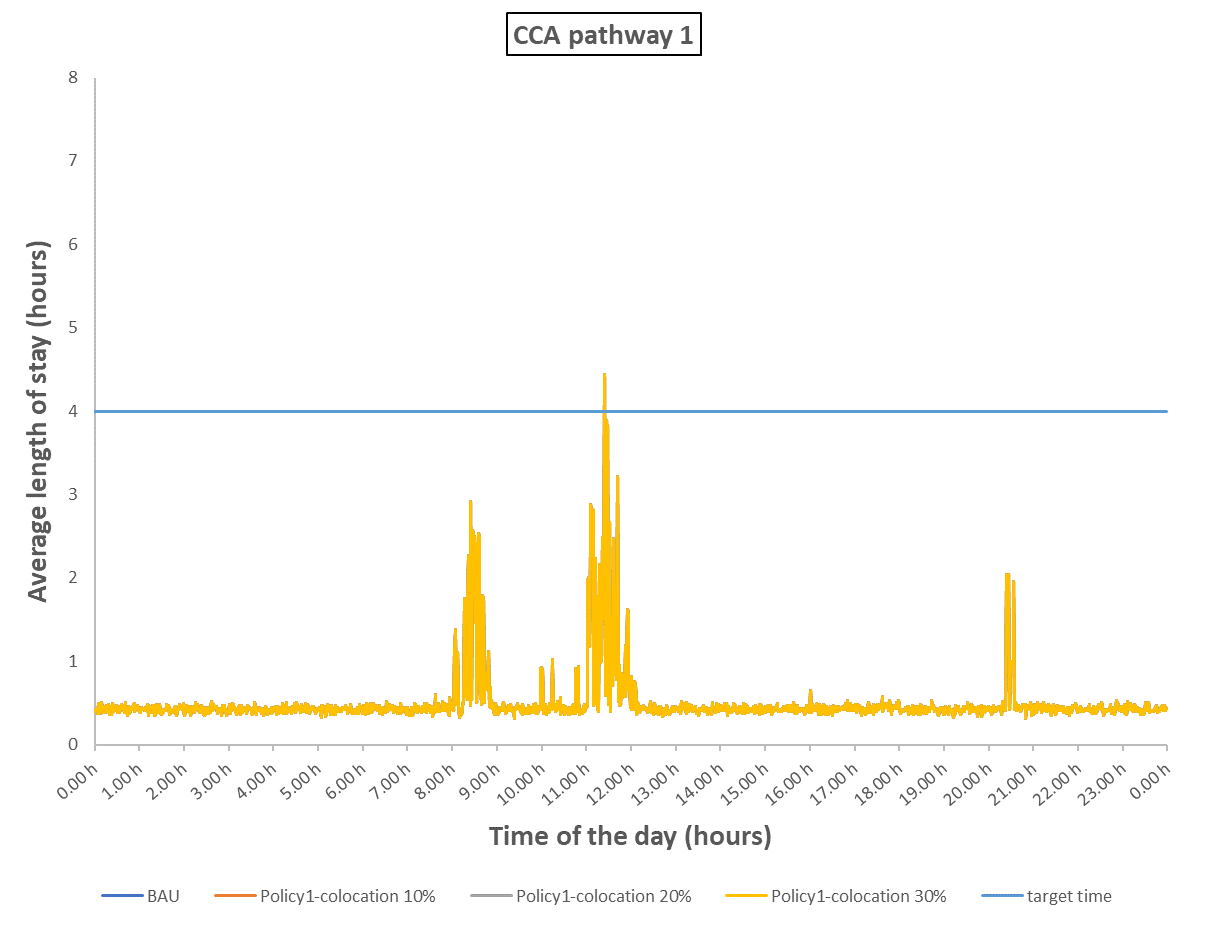 | 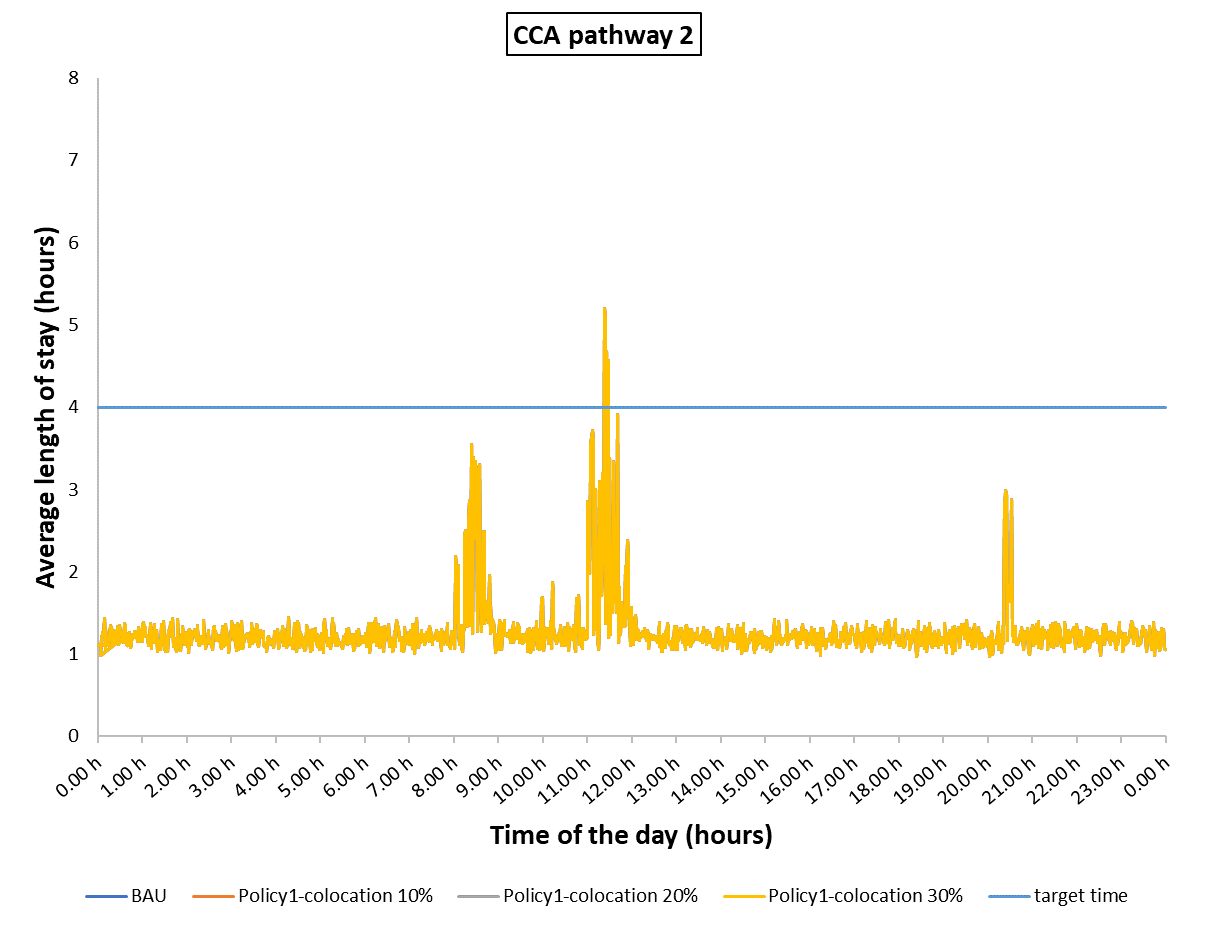 |
| --- | --- |
| 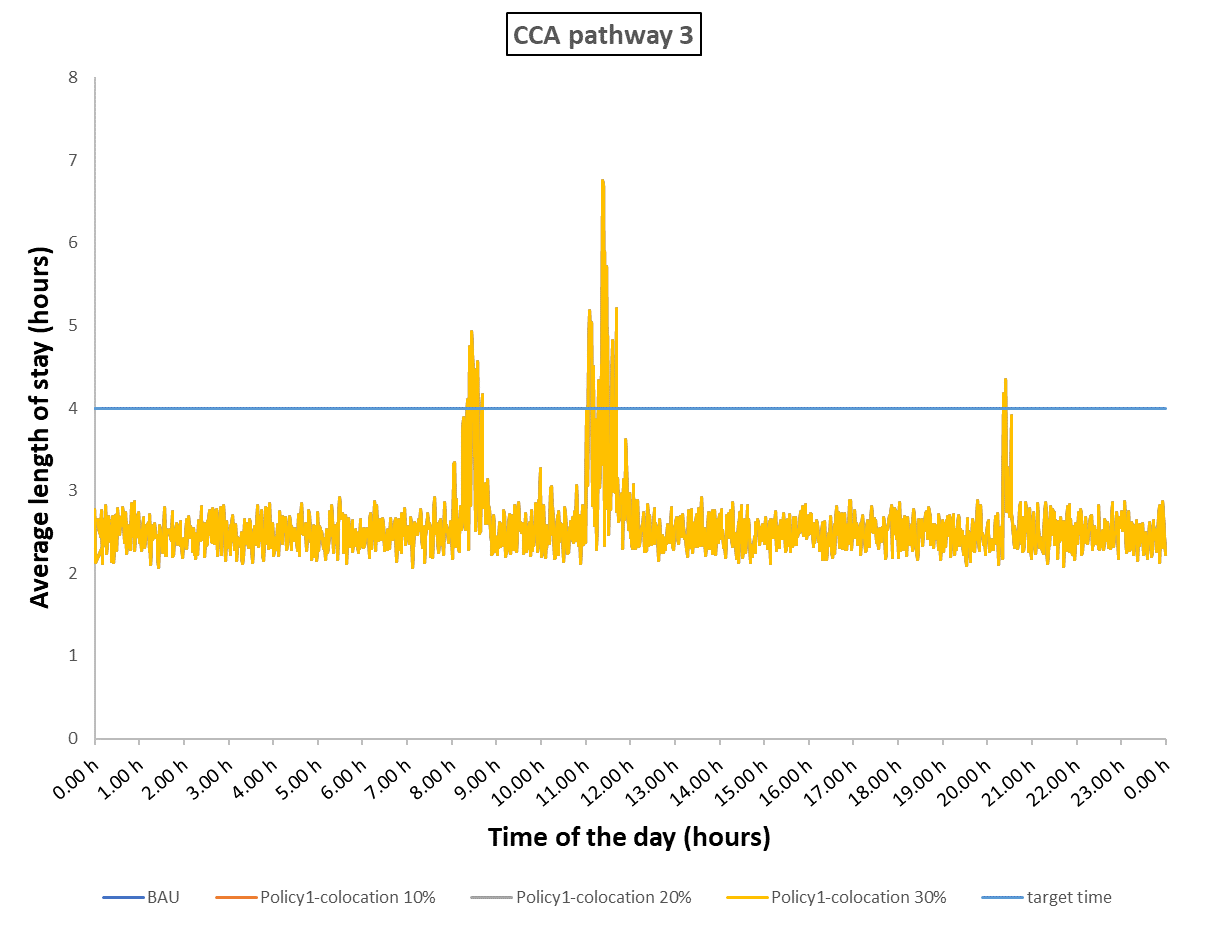 | 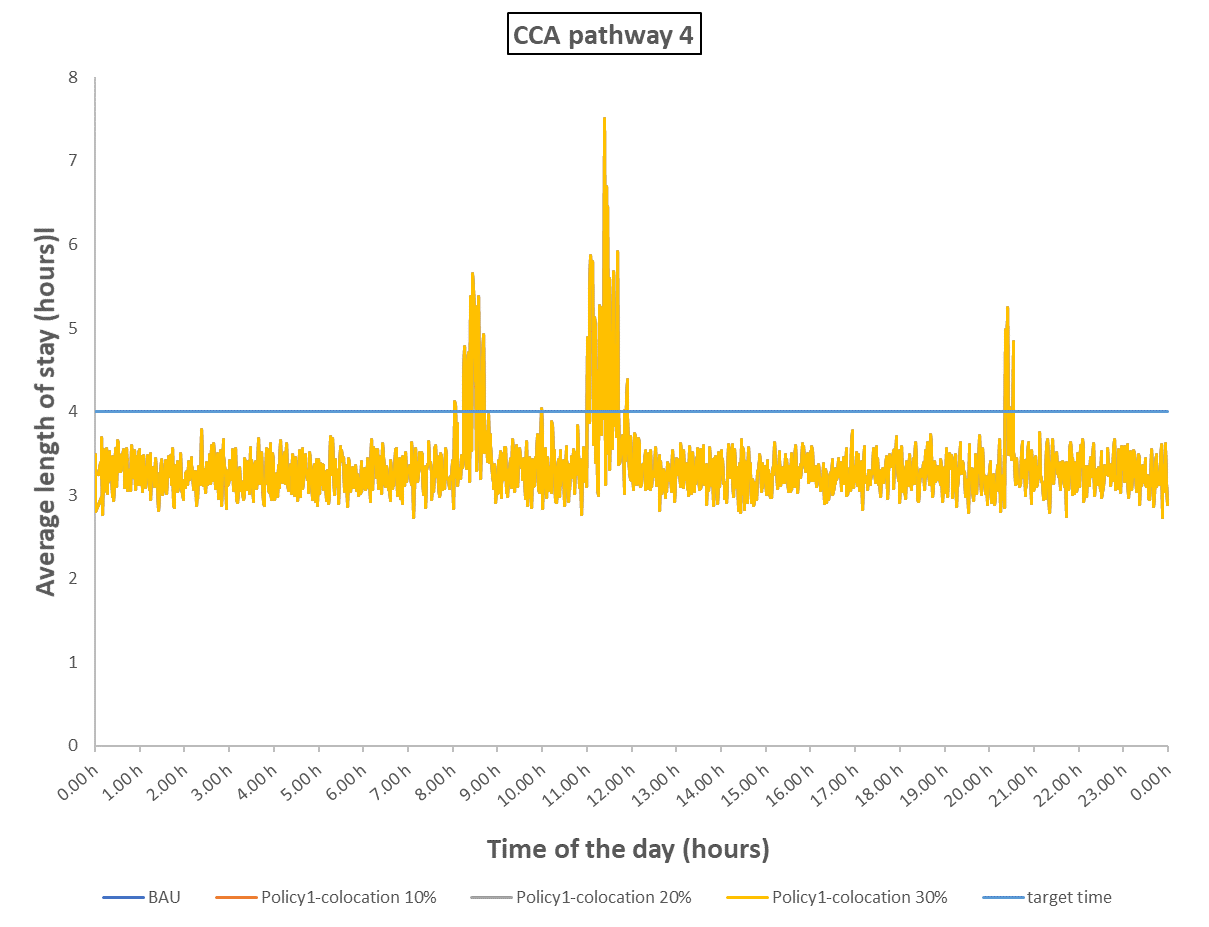 |
| 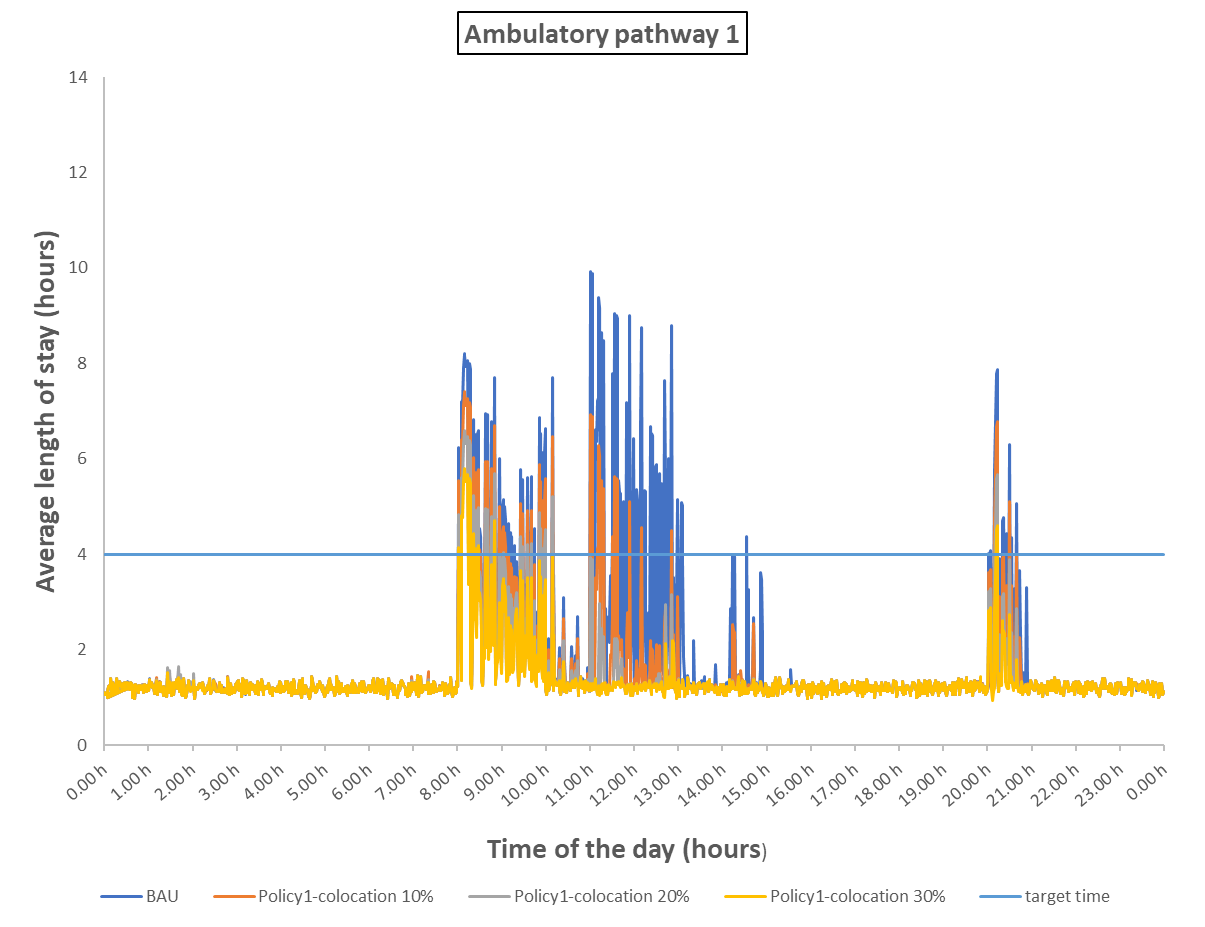 | 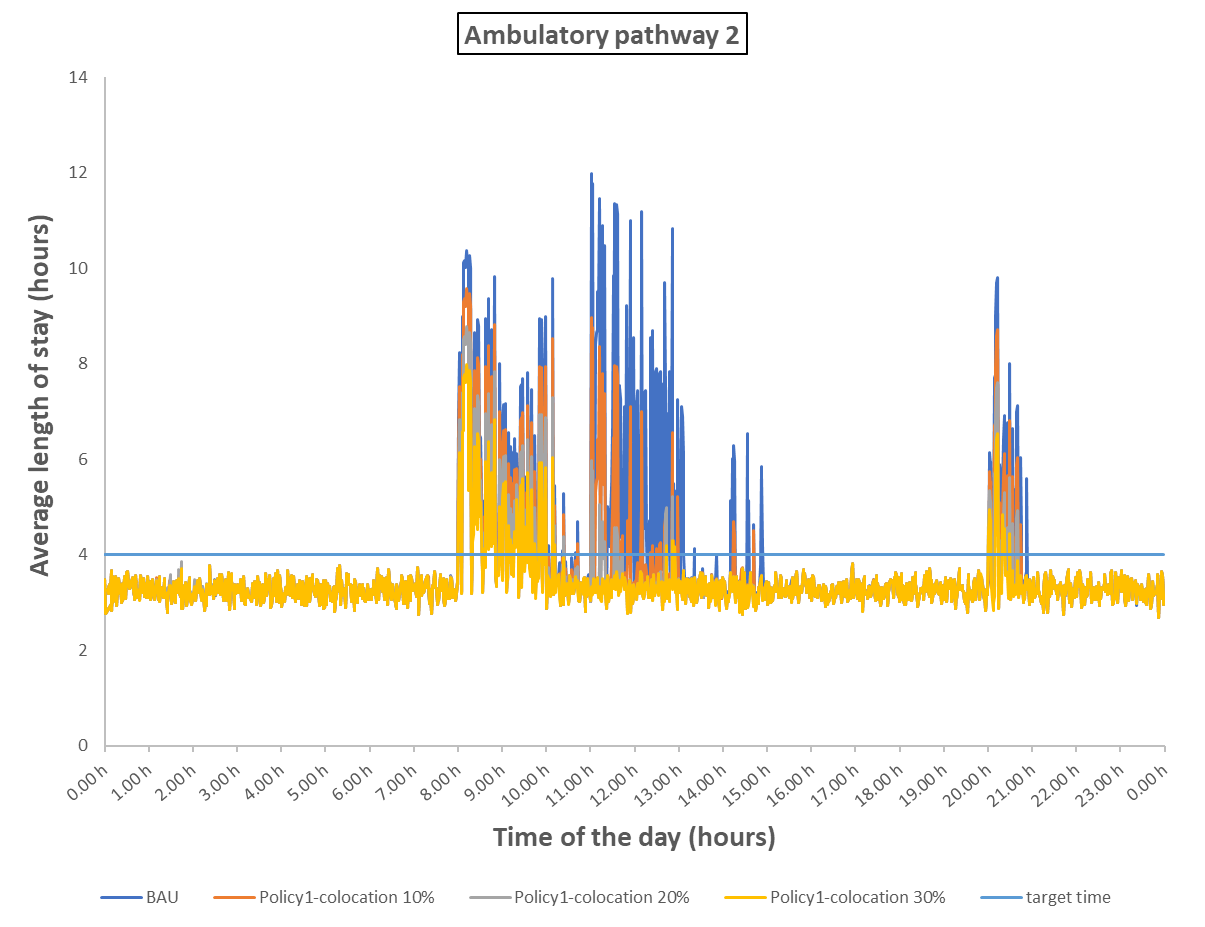 |
| 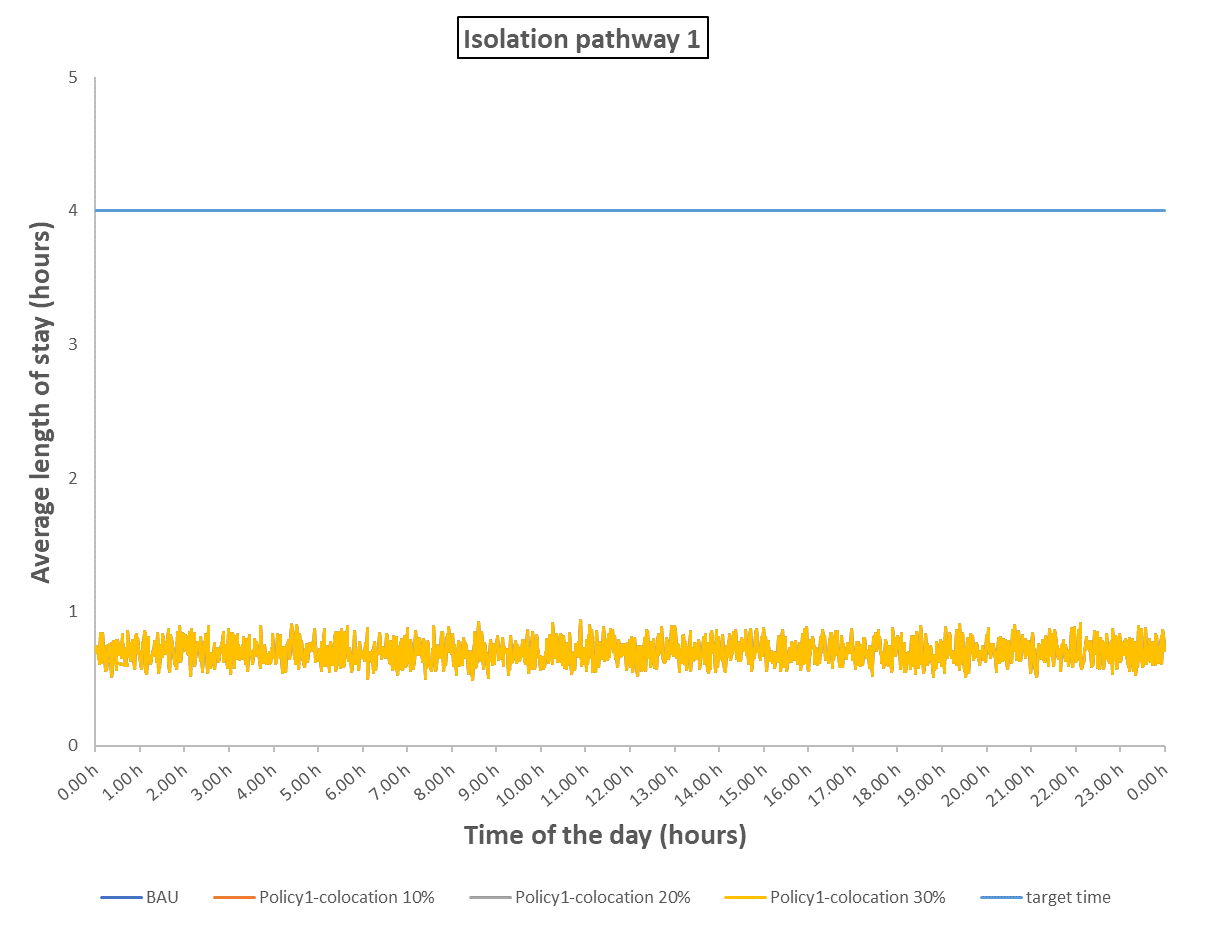 | 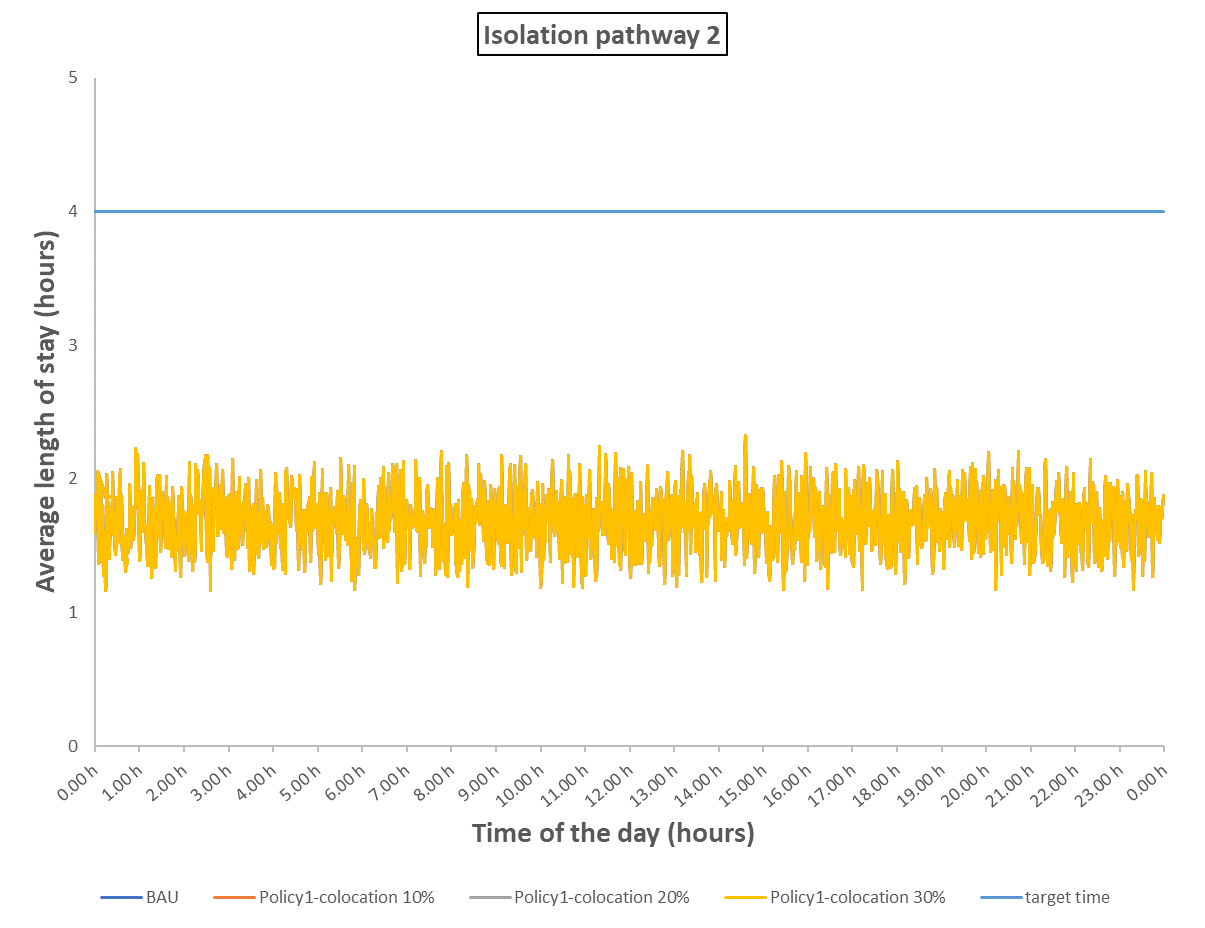 |

**S1 Fig. Average length of stay (ALOS) for ED patients depending on care venue, patient pathway, arrival time for co-location policy where 10%-30% of P4 and P3 patients are decanted from the ED to a GP clinic co-located at the ED.**
