# Supplementary material for: Modeling Emergency Department crowding: Restoring the balance between demand for and supply of emergency medicine
Source: PLoS One. 2021 Jan 12;16(1):e0244097. doi: 10.1371/journal.pone.0244097 (PMC7802975; doi:10.1371/journal.pone.0244097)
Supplement: S3 Fig — (DOCX) [file pone.0244097.s005.docx]

| 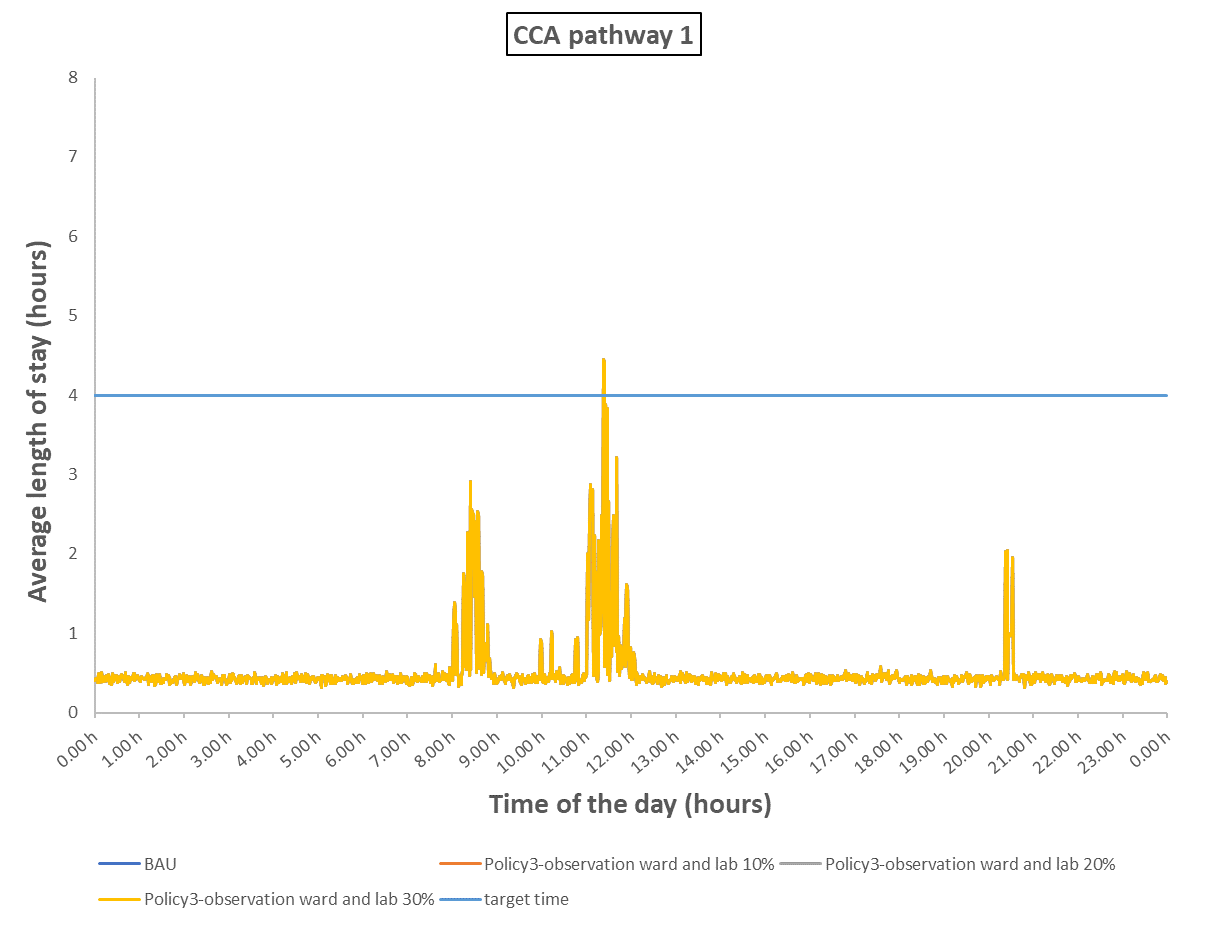 | 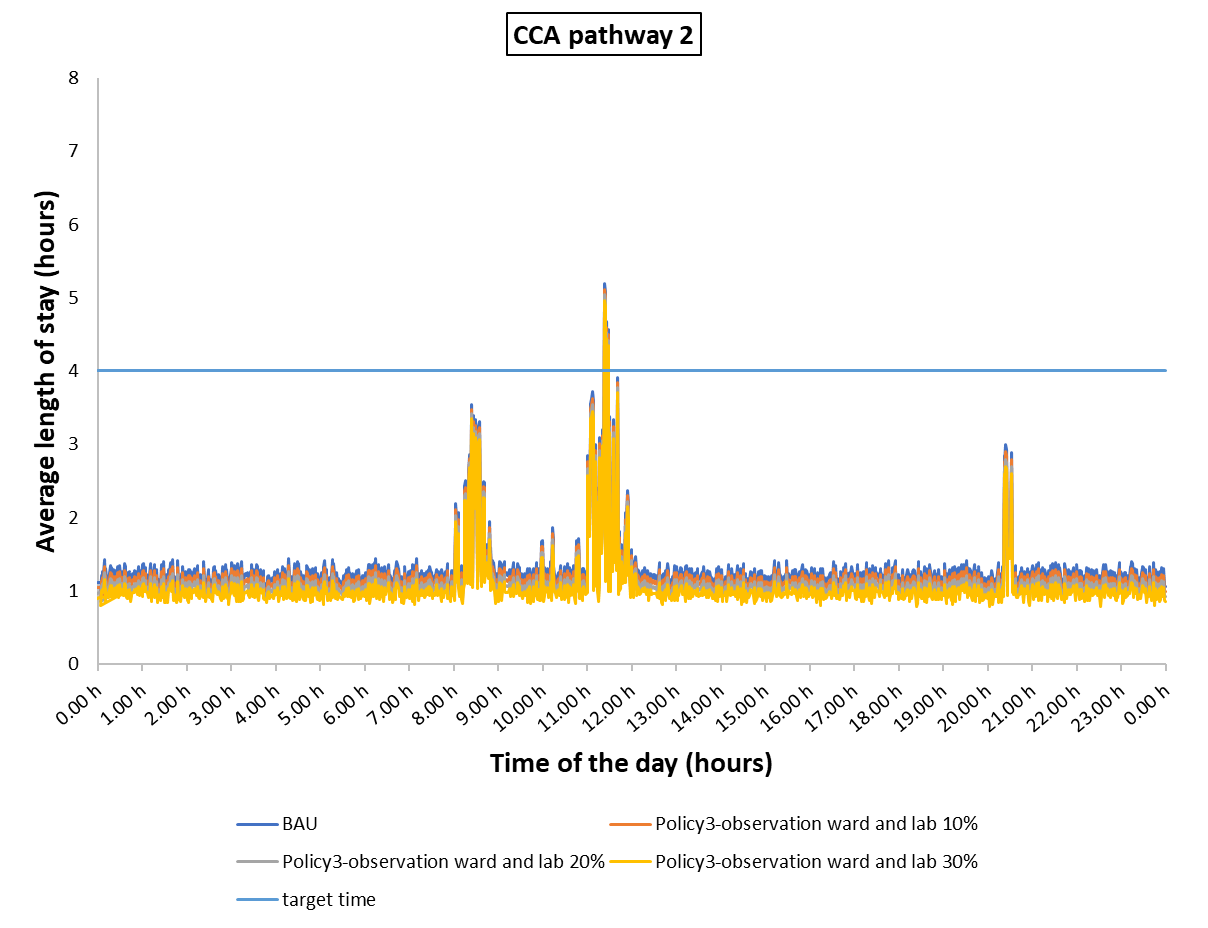 |
| --- | --- |
| 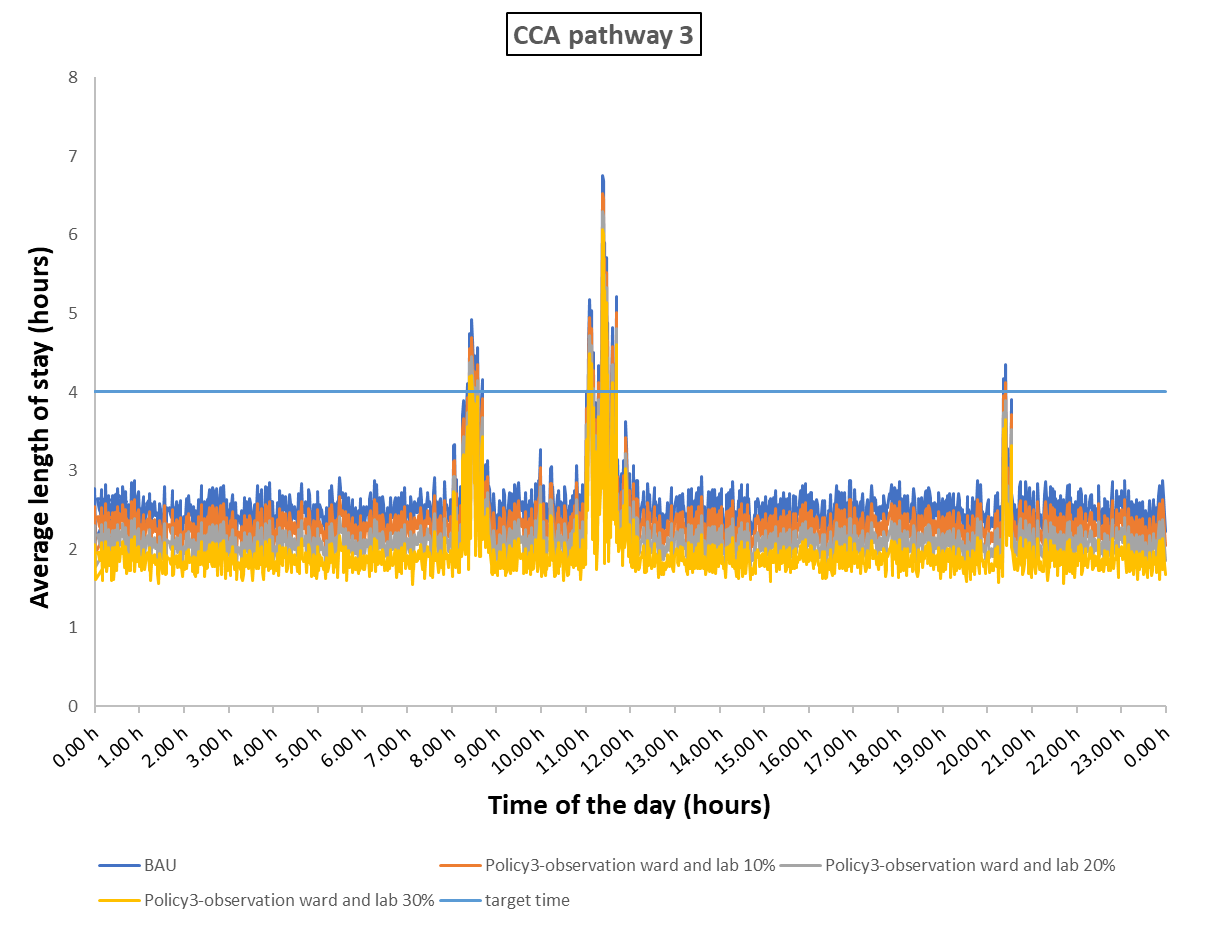 | 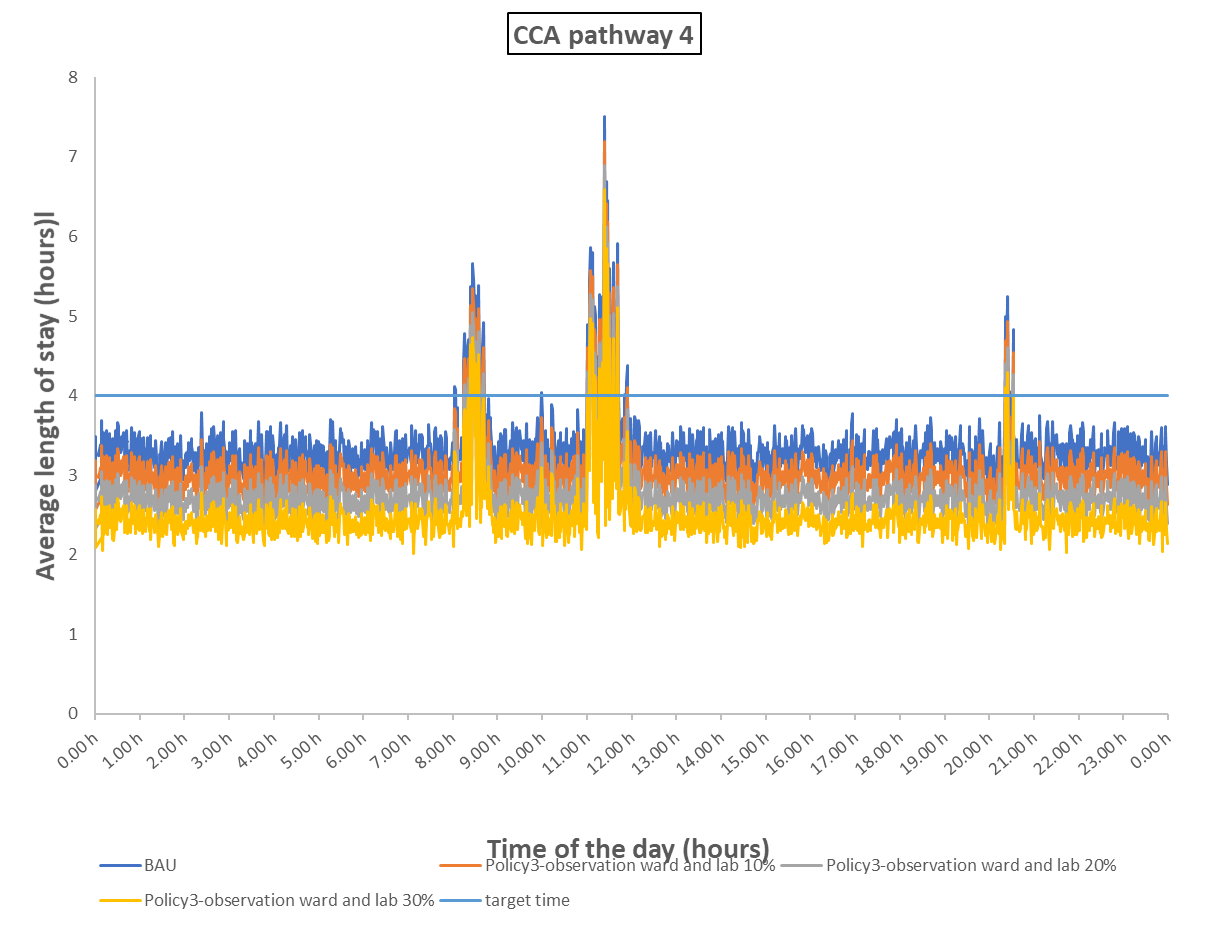 |
| 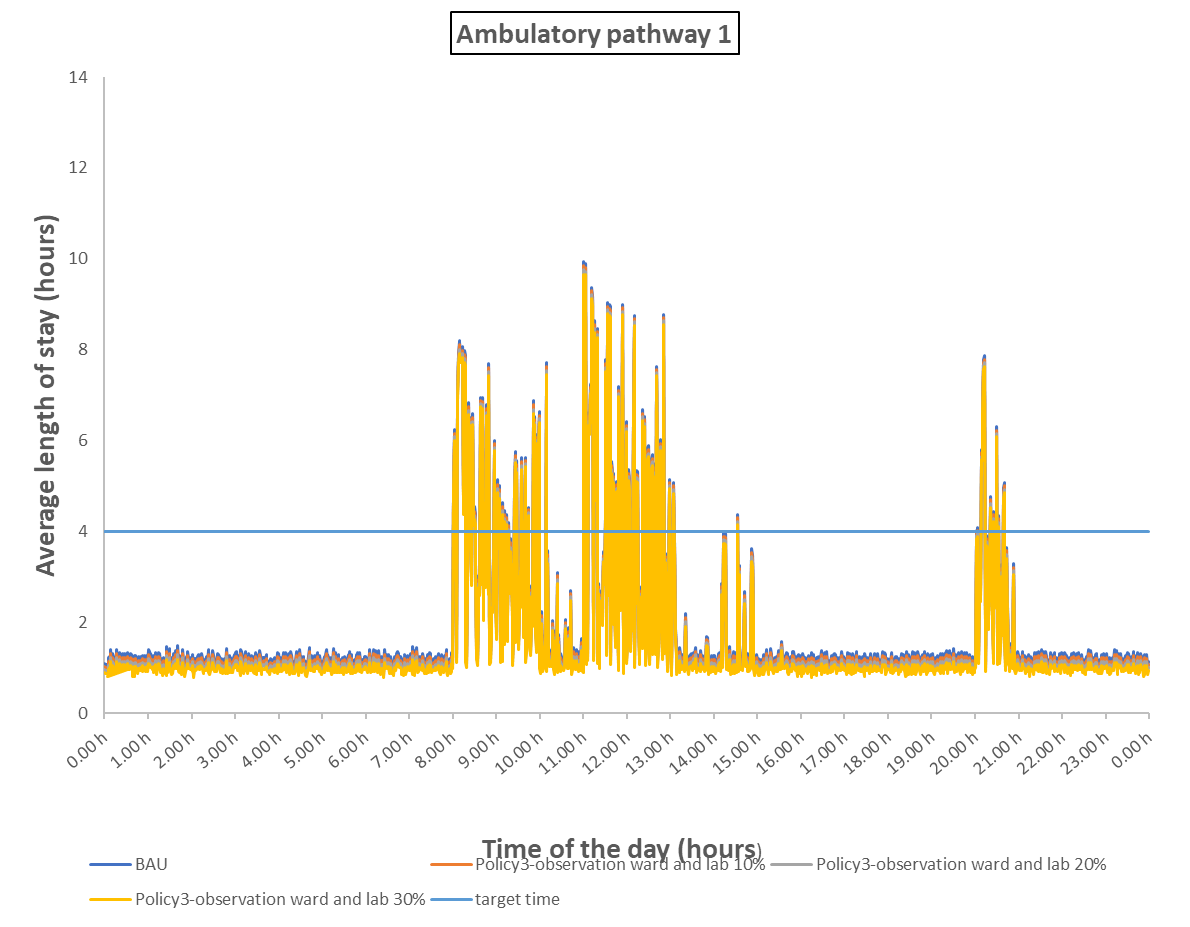 | 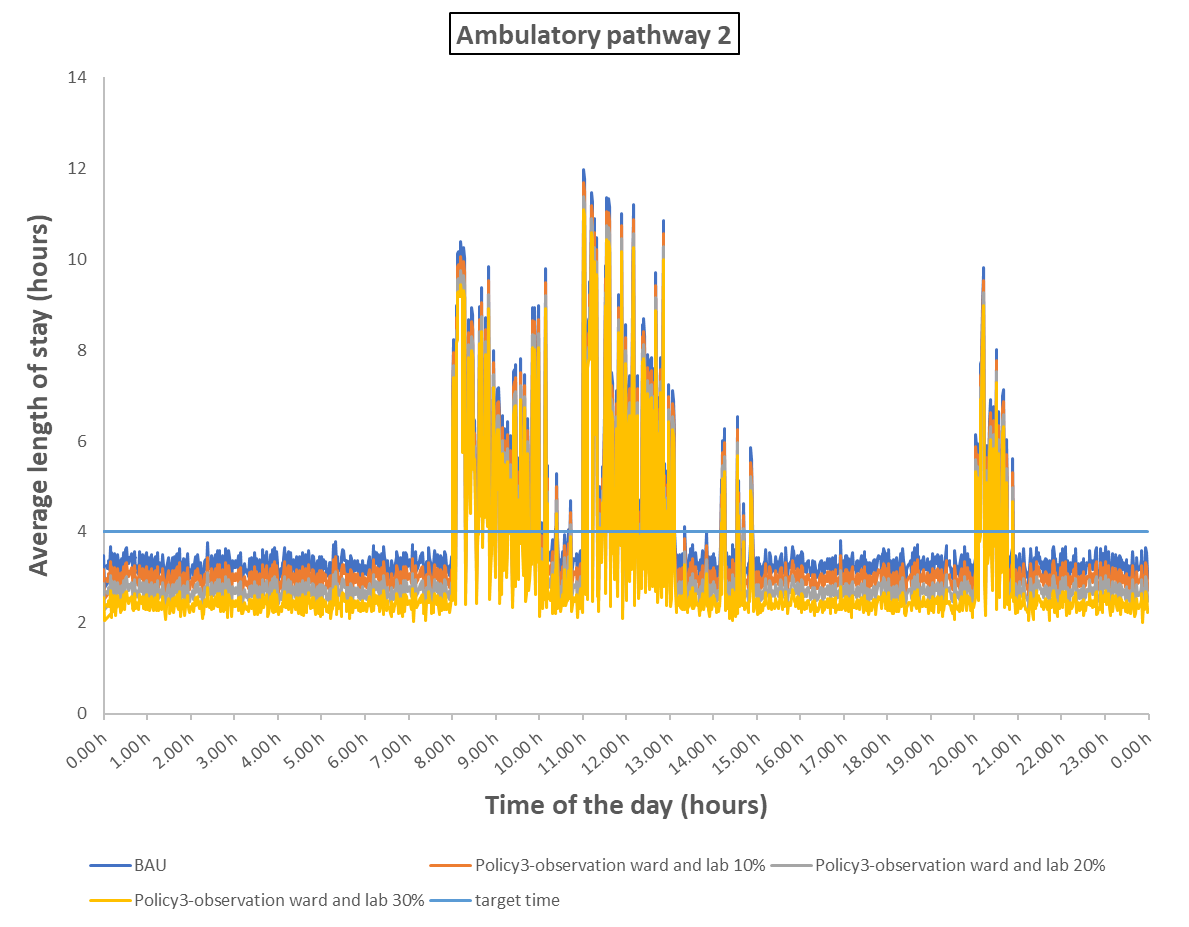 |
| 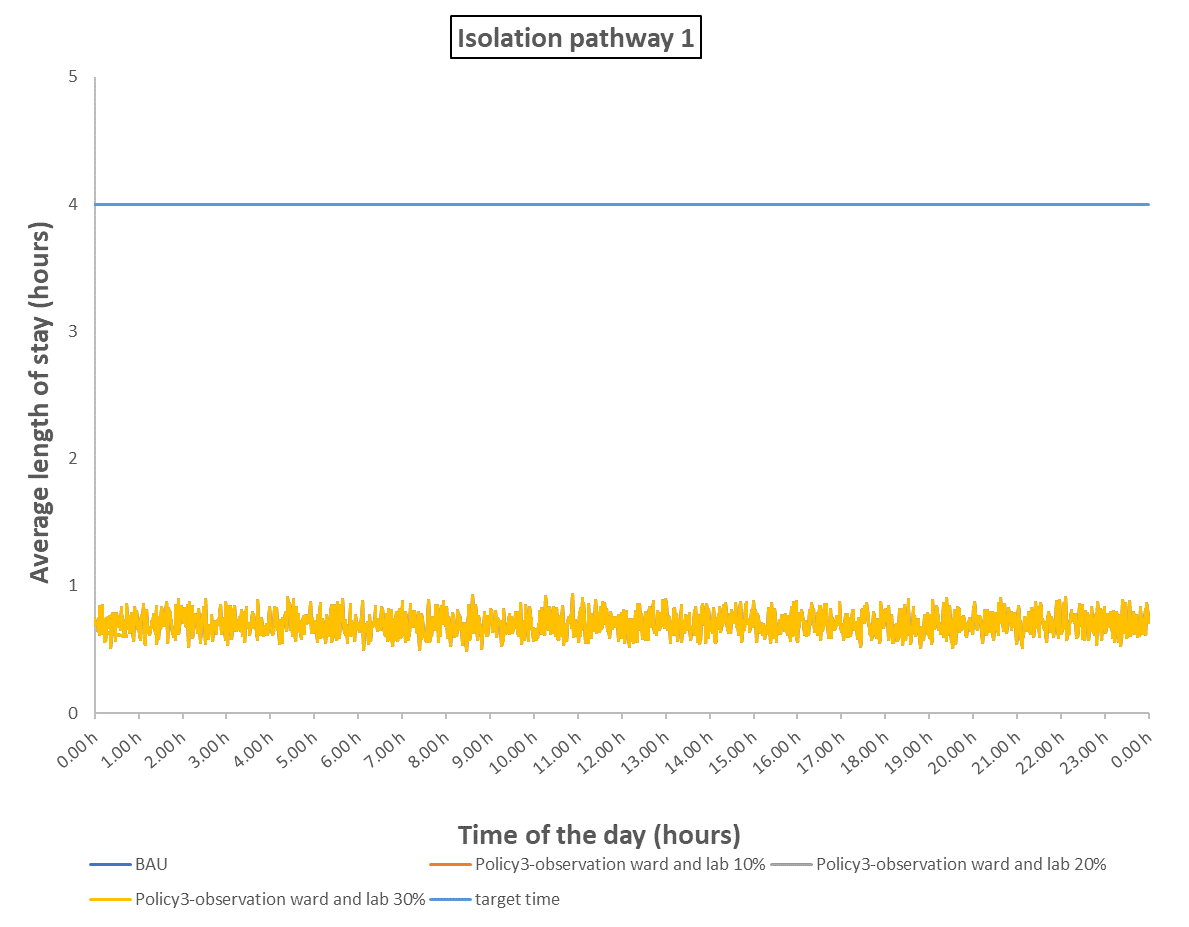 | 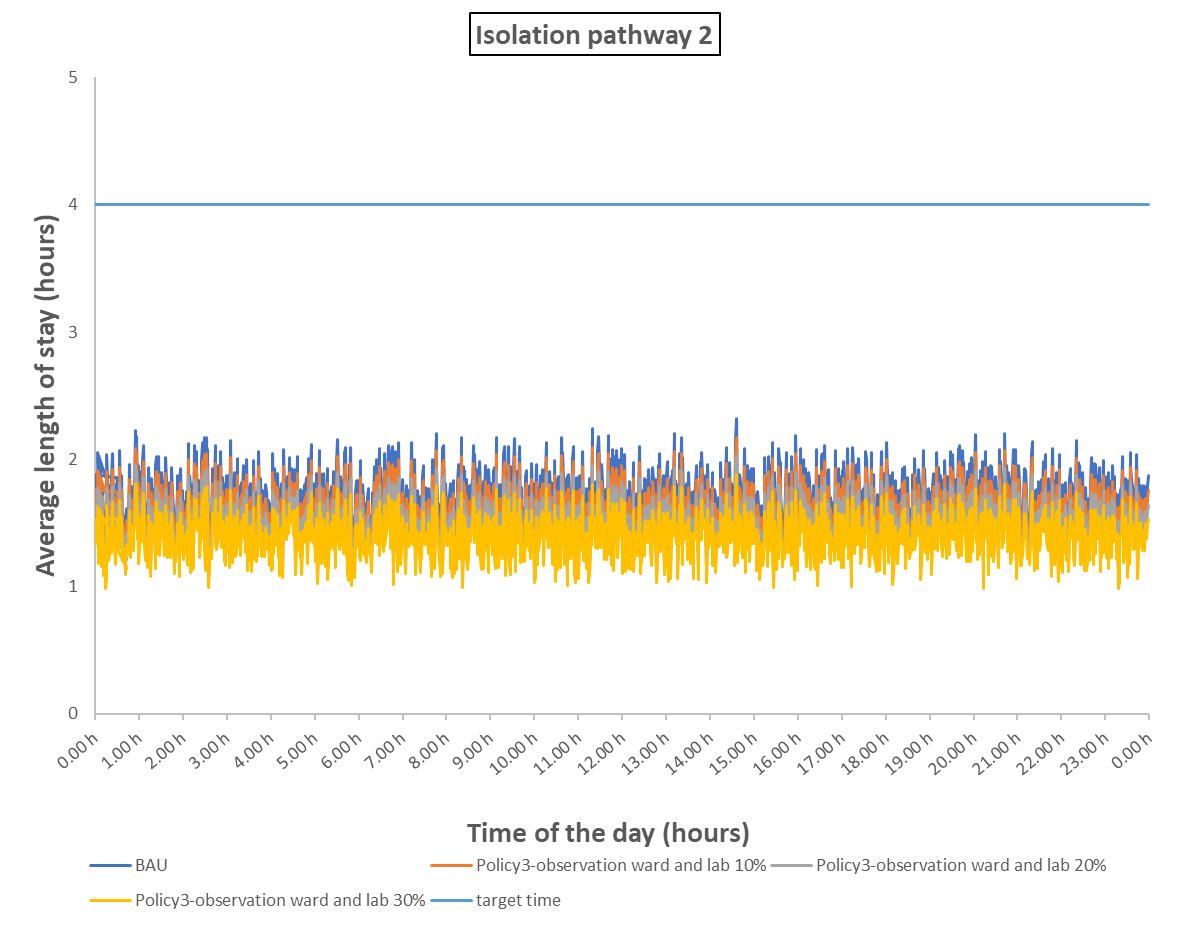 |

**S3 Fig. Average length of stay (ALSO) for ED patients depending on care venue, patient pathway, arrival time for observation ward and laboratory waiting time policy where waiting times at the observation ward, and laboratory are reduced by 10%-30%.**
