# Supplementary material for: Modeling Emergency Department crowding: Restoring the balance between demand for and supply of emergency medicine
Source: PLoS One. 2021 Jan 12;16(1):e0244097. doi: 10.1371/journal.pone.0244097 (PMC7802975; doi:10.1371/journal.pone.0244097)
Supplement: S4 Fig — (DOCX) [file pone.0244097.s006.docx]

| 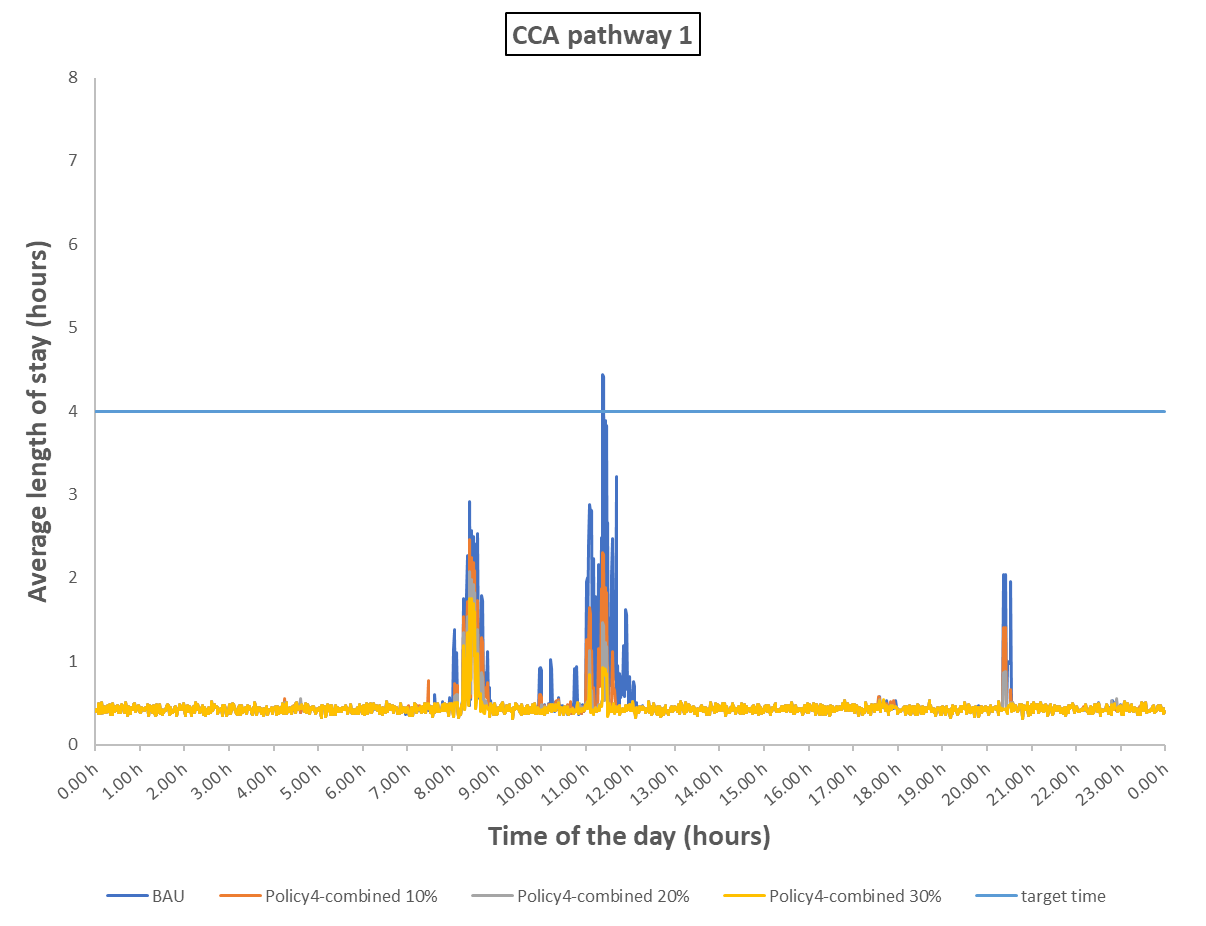 | 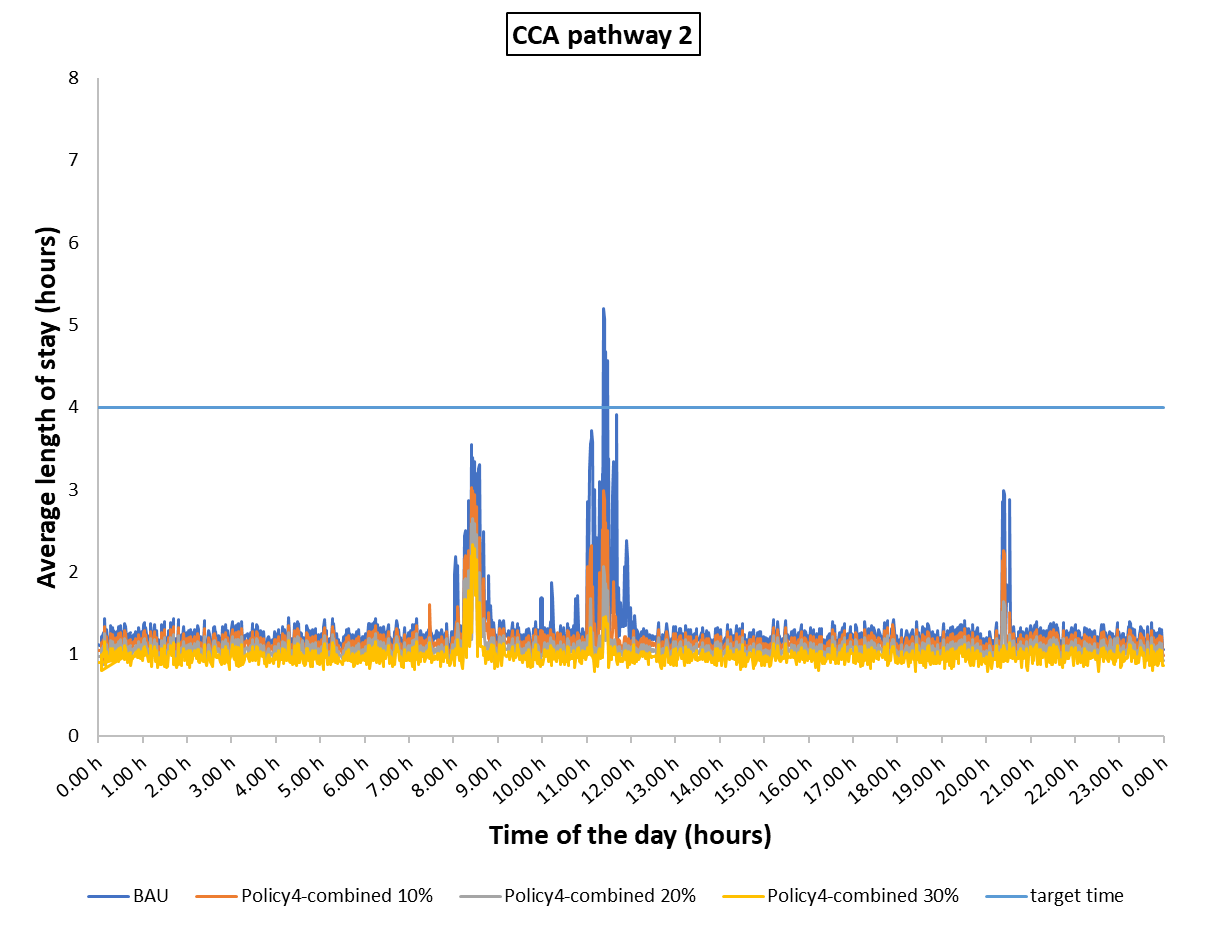 |
| --- | --- |
| 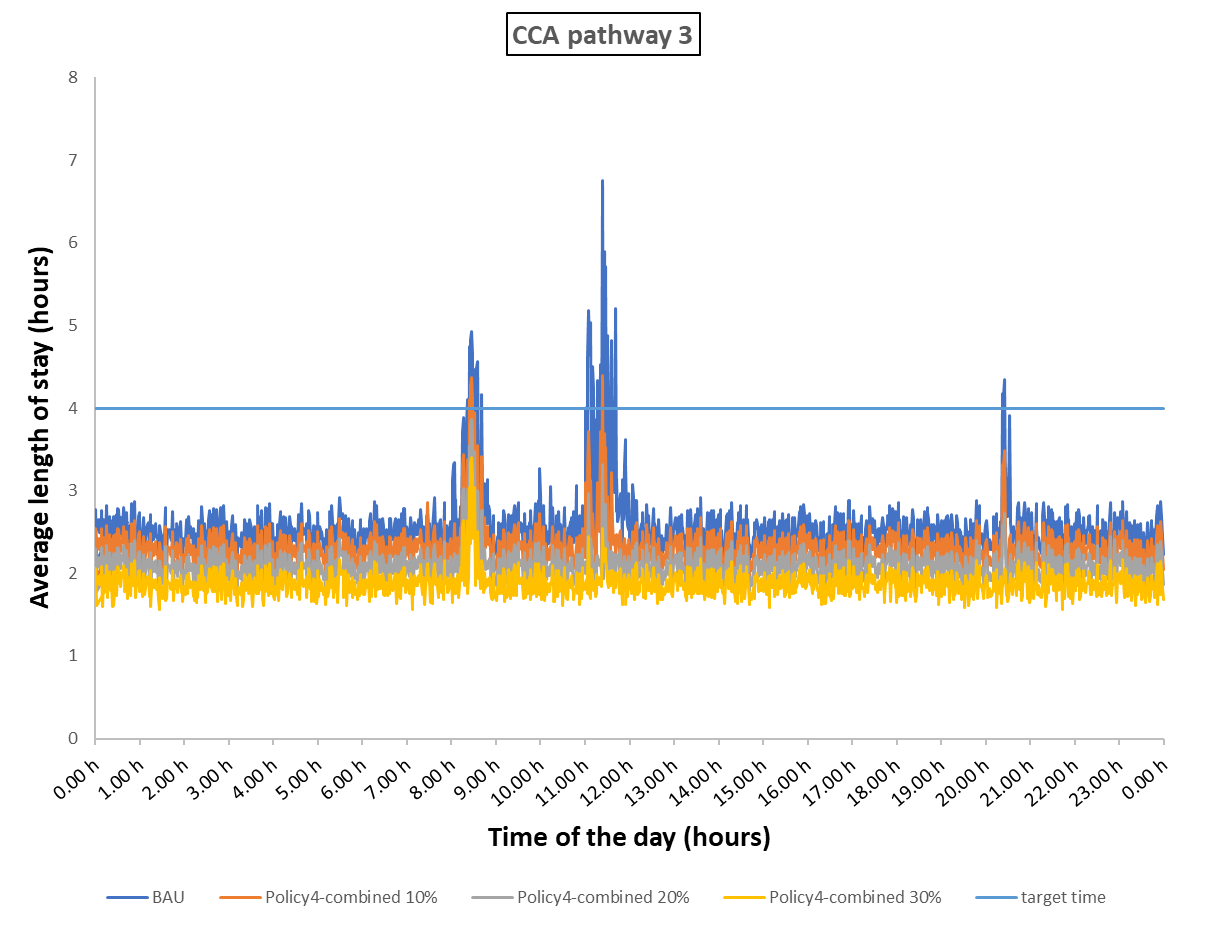 | 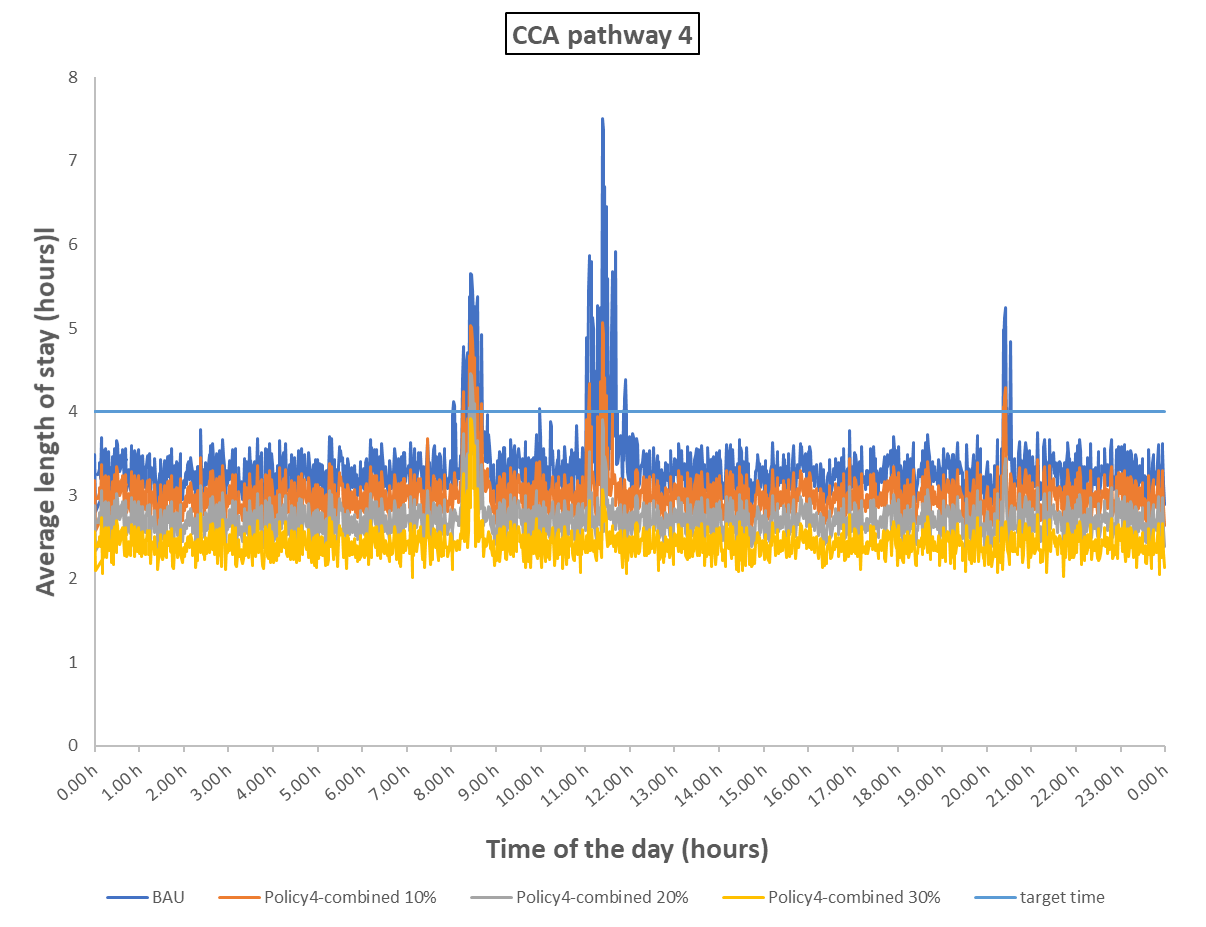 |
| 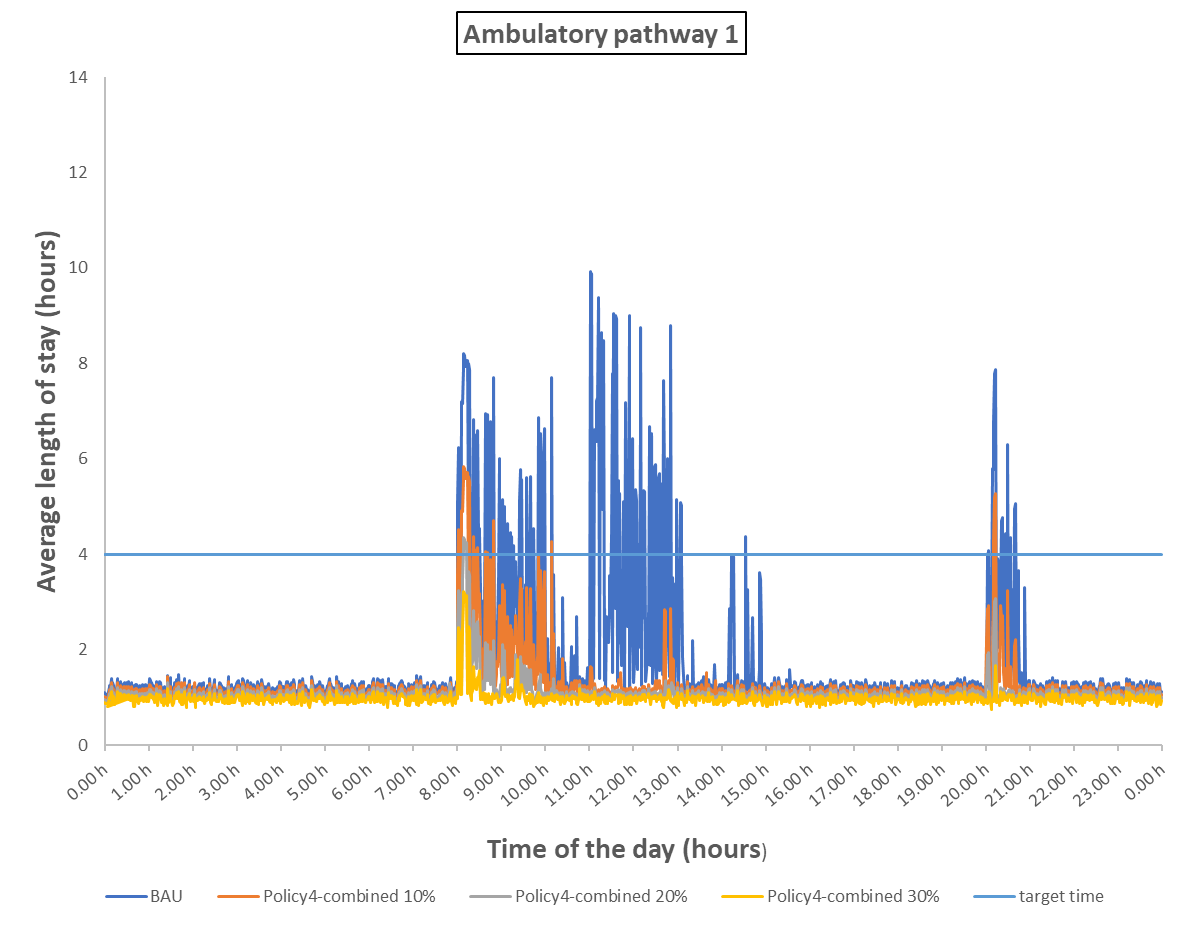 | 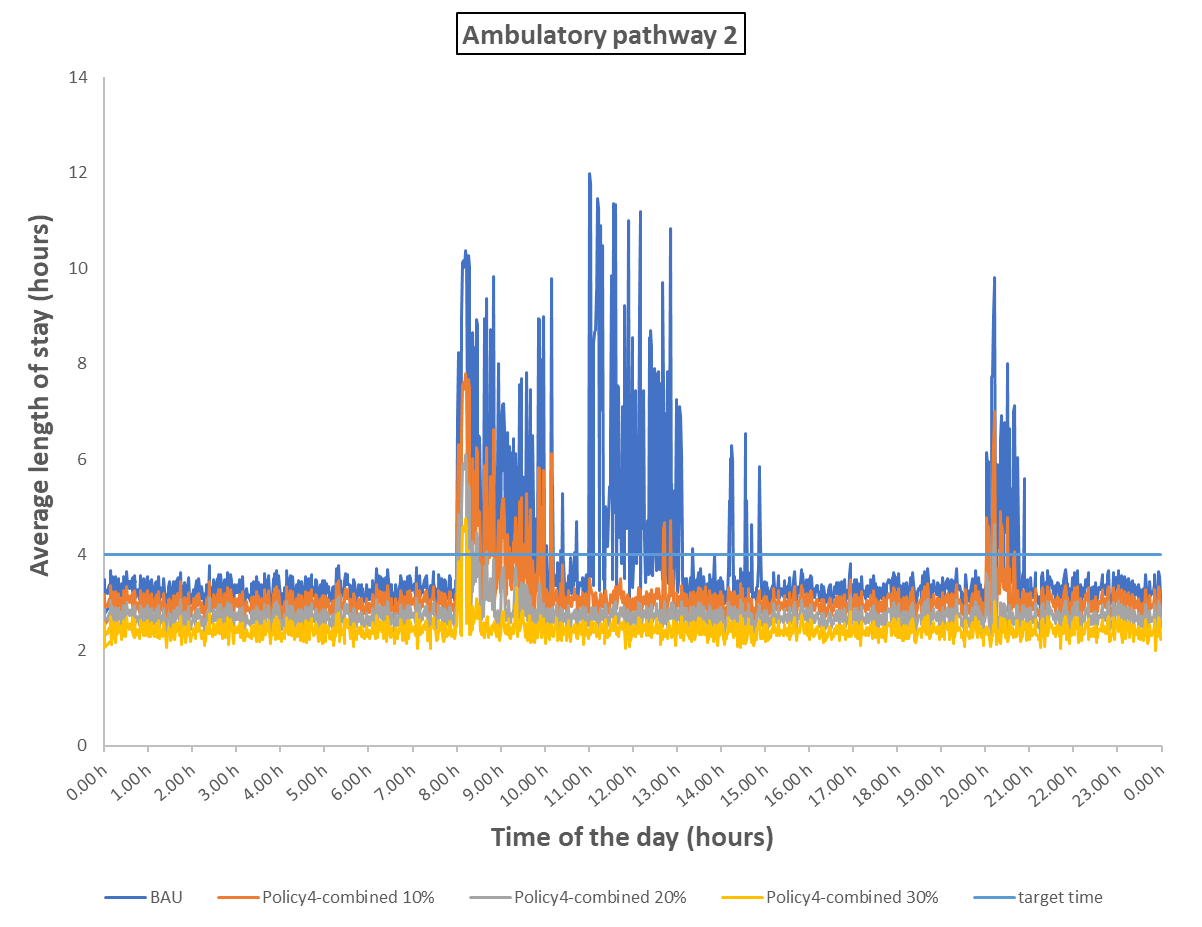 |
|  |  |
| 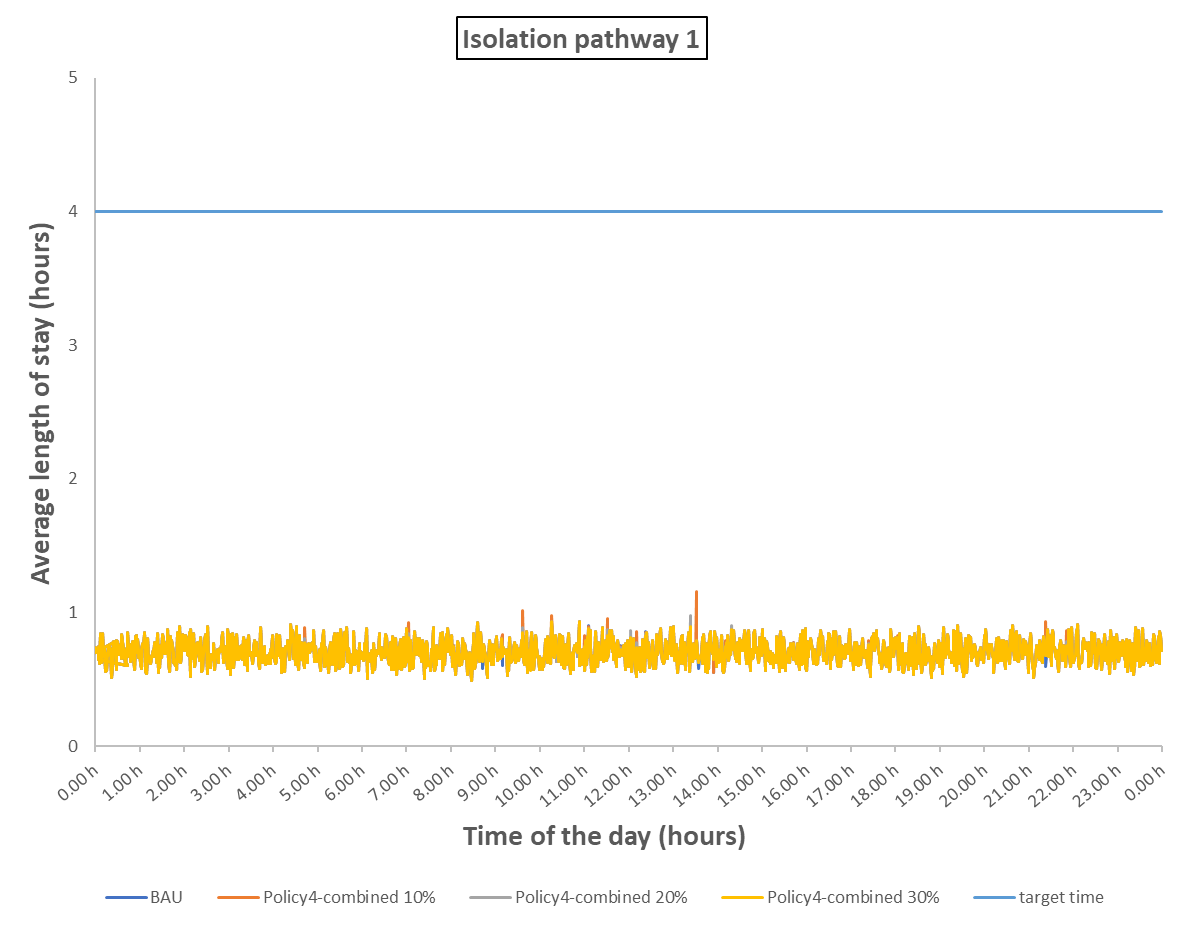 | 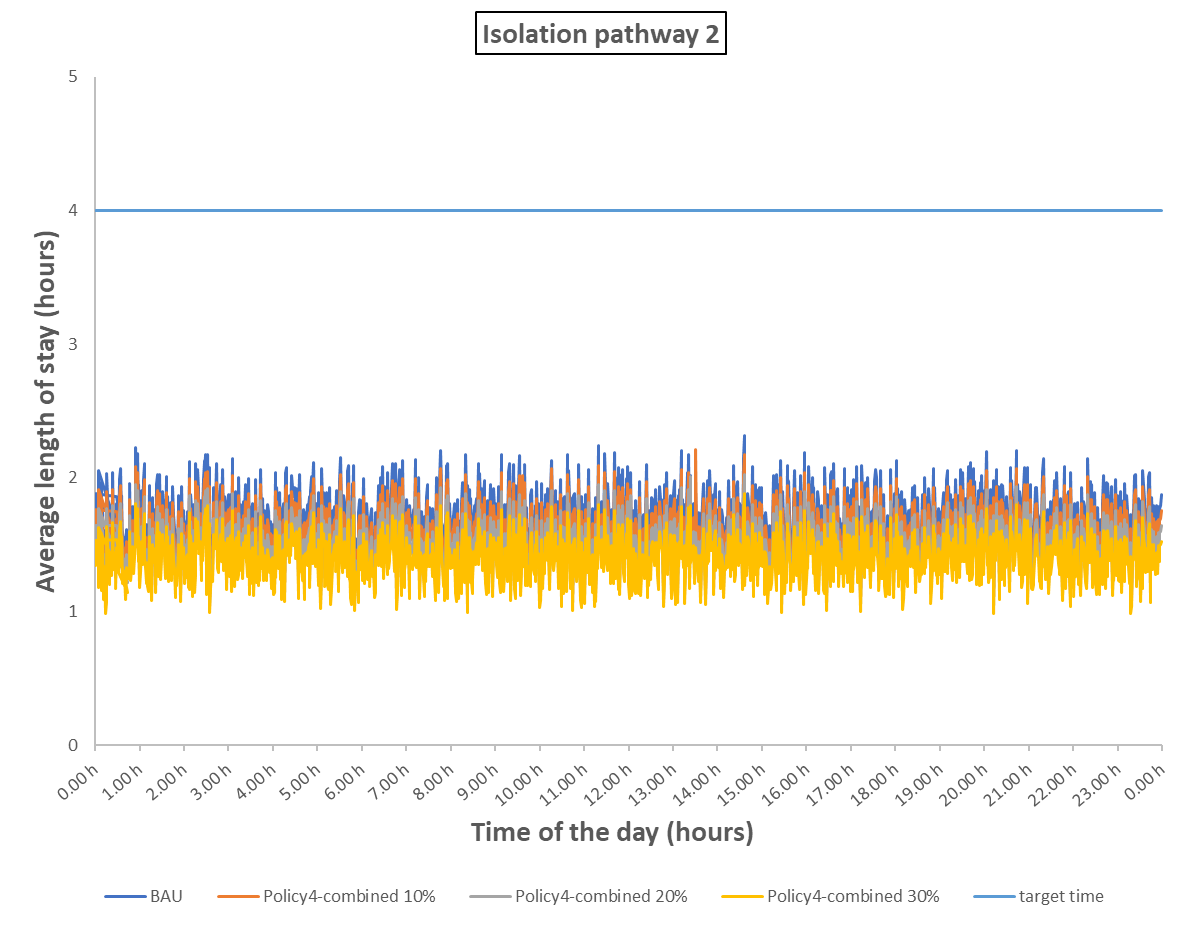 |

**S4 Fig. Average length of stay (ALOS) for ED patients depending on care venue, patient pathway, arrival time for combined interventions policy where all the interventions—i.e. co-location, capacity of doctors, and observation ward and laboratory—are implemented simultaneously.**
