# Supplementary material for: Modeling Emergency Department crowding: Restoring the balance between demand for and supply of emergency medicine
Source: PLoS One. 2021 Jan 12;16(1):e0244097. doi: 10.1371/journal.pone.0244097 (PMC7802975; doi:10.1371/journal.pone.0244097)
Supplement: Attachment — Submitted filename: Response to Reviewers.pdf [file pone.0244097.s007.pdf]

Dear editor, dear reviewers,

many thanks for your thoughtful feedback that helped a lot to improve our article. We gave our best to implement your modification requests. On a general level, we worked amongst others on the clarity of the positioning of our article in the literature, the article's contribution, the rationale and description of policies, and the way the policies are tested in the model. In the following, we comment on our changes in detail. Please note that all our changes are highlighted in **yellow color** and when we refer to page numbers, we mean the numbers in the lower right corner of each page and not the ones created automatically by the submission system.

Comments from editor		
**Nr.**	**Comment**	**Response**
1	Both reviewers also wish that the contributions of the research can be better presented. As the reviewers suggest, the literature on ED overcrowding is huge. Some recent significant related studies are suggested for review: [see proposed literature sources]	Thanks for this comment. We included more relevant literature in the paper. Furthermore, we improved the positioning of the paper in the introduction (p. 3-6). However, it must be noted that the paper clearly relates to system dynamics modeling works in the sphere of emergency medicine.
2	Please ensure that your manuscript meets PLOS ONE's style requirements, including those for file naming. The PLOS ONE style templates can be found at [see links]	Thank you for this comment. We have looked at the style requirements and adapted the manuscript accordingly.
3	We note you have included a table to which you do not refer in the text of your manuscript. Please ensure that you refer to Table 2 and 3 in your text; if accepted, production will need this reference to link the reader to the Table.	Thanks for this comment. We have ensured that every Table and Figure in the Supporting Information is also referenced to in the main text.

Comments from reviewer 1		
**Nr.**	**Comment**	**Response**
1	First, the model assumed constant sojourn time for the majority of the service processes such as waiting time (for the providers), consultation time (with the providers), time spent waiting for lab results, and time spent in the observation ward. The authors frankly acknowledged these assumptions as limitations yet provide very little justification or ramification for them. In my opinion, it is critical to capture the time- and census- dependency of these sojourn times to accurately reflect the potential improvement of a policy on the real-world ED system. It was mentioned that the authors had access to patient arrival data and conducted an additional observational study to obtain the process timings. Thus, I would suggest them to utilize the arrival and departure time stamps in the data to impute empirical distributions for the corresponding sojourn times. This effort, if feasible, should increase the robustness and credibility of the results.	Thanks for the comment. We have changed the constant sojourn time with an empirical distribution for the corresponding sojourn time to increase the robustness and credibility of the results. We used triangular distributions to represent our empirical distributions in the simulation.
2	Second, there seems to be room for improvement for the policies examined. To be more specific, Policy 2 imposed a 20% reduction on boarding time (time spent in the observation ward waiting to be transferred to the main hospital). I wonder where this value comes from – whether it is realistic, and if so, how it can be achieved. Also, instead of presenting an isolated value I would recommend testing a few more scenarios, e.g., from 5% to 50% with 5-10% increment. Although I hold doubt about the feasibility of reaching a time invariant reduction in boarding time, it would be great to see some explanations for the choice of this value, as well as some concrete examples of how it can be achieved in a real ED system.	Thanks for the comment. We have changed the policy implementation approach taking into consideration your very important suggestion. Thus, all policies are now implement using a scenario where the policies are changed with 10%, 20% and 30% to explore their impact on average length of stay (ALOS).

3	My concern with Policy 3 can be summarized in a similar vein. I.e., there seems to be a lack of rationale behind the single choice of 20% for the increase in physician capacity. Moreover, the authors addressed this policy as dynamic. I could be missing something here, but it seemed to be a static policy in that the staffing capacity was not dependent on the system state (time or census level). It would be interesting to see the authors incorporating a few more sophisticated policies targeting at unclogging the bottleneck of the queues, as well as providing some discussions around how the target reduction of demand or increase of supply can be obtained in reality	Thanks for the comment. First, we have changed the name of the policy from "dynamic doctor allocation" to "optimal doctor allocation". Second, we have changed the implementation of the policies to ensure consistency. Each policy is implemented under three scenarios i.e. 10%, 20% and 30%, to explore it impact on ALOS. In addition, we have included in the discussion how these policies could be achieved.
4	Add a section for input parameter analyses and present estimated values for the key processing times such as triage time, registration time, waiting time to see a physician, consultation with a physician, waiting time to be transferred, etc.	Thanks for the comment. We have added a section on input parameter in the methods section to present the key processing times.
5	Provide definition of AT (adjustment time) in the paper and short explanations for its use in the equations	Thanks for the comment. Adjustment time is a model artifact to ensure unit consistency. The value is 1 and it does not have any impact on the equations used but to ensure unit consistency.
6	In my humble opinion, it would be sufficient and possibly serve better visual purpose to present smoothed hourly average ALOS instead of the histogram of raw simulation data in Figure 10 and 11.	Thanks for the comments. We have moved the graphs to the supplementary file.
7	In Figure 10 and 11, it would be good to use more distinctively different colors for base case scenario and target time horizontal line.	Thanks for the comment. We have improved the colors used to ensure clarity
8	Since in some scenarios policy 1 and 2 had no effect on reducing ALOS it would be less confusing to use the same legend or add a footnote to the bottom of Figure 10 to highlight this part of the results.	Thanks for the comment. We have taken that into consideration.

Comments from reviewer 2		
**Nr.**	**Comment**	**Response**
1	Reading the Abstract, the actual contribution and main conclusion of the paper is not clear. The authors should explicitly state the novelty and main insights of their work	Thanks for this comment. We have expanded the abstract to include the new policies that we have tested and to be clearer on the main conclusion of the article
2	The Introduction section fails to clarify the academic and practical contribution of the paper. What has already has been done in the field? How does this paper differ from existing research? What is its specific added value, both for theory and practice?	We have adapted the introduction on p.5-6. We tried to be clearer on how we position the article in the body of literature. The purpose of this study - as in any system dynamics works - is twofold: (1) create a simulation model of a dynamic phenomena under interest; (2) test relevant policies to alleviate the negative consequences of the dynamic phenomena under interest. In this case, (1) the model is a detailed representation of a hospital-based ED in Singapore. The model is a comprehensive representation of all major patient flows (demand side) and medical resources (supply side) in the ED. Furthermore, we completely reveal the entire structure of the model plus its mathematical formulation. To the best of our knowledge, such a model has not existed in the literature so far. (2) We analyzed policies that are currently being discussed in Singapore as potential candidates to relieve the burden on emergency medicine. We have used system dynamics (SD) as our modeling method because SD is a very natural method to model stocks of patients and patient flows considering that its main modeling elements are "stocks" and "flows". Additionally, SD has a long history of successfully involving clients/stakeholders in the modeling process. Due to its graphical interface and its easy to understand graphical iconography SD lends itself to co-create models directly with stakeholders - a capability that is hard to match with other modeling methods (discrete-event simulation, agent-based modeling etc.).
3	p2, last paragraph: “Typically, patient arrival patterns are cyclic … This variable demand poses an additional burden on ED management teams.”. The fact that arrival patterns are cyclic doesn’t necessarily result in a more difficult management of EDs, as the same pattern can be repeated every time. However, in addition to the cyclic pattern, patient arrivals are unpredictable and stochastic, which makes ED management more difficult.	Thank you. We changed this sentence to reflect this fact (see p. 3)

4	p2, last paragraph: "Although the number of emergency physicians (EPs) has risen, that is, 13.4% annually…". Can it be explained why there is a high physician workload and ED crowding, while the number of ED physicians has risen more than double as fast as the number of ED patient visits? One of the investigated policies focuses on the capacity of ED physicians, but are they really the bottleneck resource?	Thanks for this comment. We suspect that there is still a mismatch between the size of the staff and the demand for emergency medicine services. It is true that the number of emergency physicians has risen faster than the growth in demand, but considering the massive workload of EP's still points towards an undersupply of personnel
5	Figure 2: The word "quite" should be replaced by "quiet" in the caption.	Thank you. We changed the word in the caption (p.4)
6	p4, first paragraph: "This allows identifying bottlenecks in the system, i.e., ED venues where patients accumulate and put a strain on ED performance.". The authors define bottlenecks as areas in the ED, rather than specific ED resources. However, resolving a bottleneck which is a complete area in the ED is difficult to accomplish and requires further examination of the specific problem areas within the venue. In addition, this paragraph suggests that bottlenecks will be identified by means of the simulation model, which is not the case in the paper.	Thanks for this comment. We dropped this paragraph completely.
7	p4, first paragraph: The description of the three investigated policies is a little bit misleading. For example, it is stated that the paper will investigate "How ED manpower needs to be adjusted in order to dissolve congestions and smooth ED patient flows", while only a 20% increase in physician capacity for all venues at all times is investigated, without a prior identification of the bottleneck resources/venues/times and accompanying required adjustments in ED manpower.	Thanks for this comment. We changed the description of the policies to make clearer what the intentions behind are (see p.5 and p.26-27)

8	p4, last paragraph: "To the best of our knowledge, there is no study using SD on the development of a virtual ED, as we understand the term—a simulation model that comprehensively reflects all major patient flows and medical resources in an ED—that is fully transparent (documented) and accessible for researchers and subject experts.". Is this the main contribution of the paper? And to what extent is the developed simulation model generic and reusable by other researchers, as the structure of the simulation model is based on the ED under study (e.g. care pathways, venues…)?	Thanks for this comment. As previously explained, this is one of the key contributions of this article. We have integrated the sentence you reference to into the paragraph relating to the purpose of this article (see Introduction p. 5) The model is indeed generic in that it can quite easily be translated into any other hospital-based ED located in an urban area. Currently, we are adapting the model to fit to the largest ED in Switzerland. From our experience, we have observed that almost any larger ED in an urban setting worldwide offers some basic functions (e.g., "critical care" (resuscitation care), "ambulatory care" and "isolation care")
9	Only six studies in the vast amount of ED simulation literature are described, while the authors indicate that a recent literature review paper (Salmon et al., 2018) identified 18 studies that applied system dynamics to EDs. Why only focus on these six studies? What is the link/difference between the described studies and this paper? What are the shortcomings of these studies and how are they dealt with in the current study?	Thanks for this comment. A thorough literature review is neither the purpose of this article nor is it in the scope of this article. We focused on some key system dynamics works related to modeling emergency care processes. As having more than a decade experience in modeling emergency services with system dynamics we know quite well the important literature that has been published in this domain. Furthermore, as explained previously our developed model is unique in its comprehensiveness and transparency and clearly fills a gap in the SD literature on modeling emergency medicine services.
10	Some references are wrongly placed between brackets in this sections, e.g. (Lane et al., 2000).	Thank you. We changed the respective references (see p.6/7)
11	p5, first paragraph of Literature Review section: "… (the rationale for choosing SD is explained in the study setting section below)". As this is the second time the authors refer to the study setting section for the rationale behind choosing SD, a short explanation can already be provided here to justify this choice and to clarify the contributions compared to existing literature. Why is it important to use SD in addition to all DES studies? What are the advantages?	Thanks for this comment. We dropped this sentence and provide instead a very quick justification of method selection it the Introduction (p.5)

12	It is not clear how this paper contributes to the existing (and large) body of literature. What is the contribution to existing literature and what is link between existing literature and this research? In case the main contribution of this paper lies within the use of system dynamics to model an ED, a more thorough discussion of the advantages of SD, existing SD models of EDs, and the novelties within this model is required (see previous comment). In case the main contribution are the investigated policies and accompanying insights, an overview of existing studies that examine the three policies investigated in this paper can provide useful information on the added value of this paper compared to existing studies.	Please see our answer to comment 2.
13	From the Methods section (Section 3.1), it does not become clear what the advantages of SD are. Why is it important to also use other methods than DES, and especially SD? Which new insights can SD provide? The use of a diversity of simulation methods is only valuable when all the different methods have their advantages (which is the case, but does not become clear from the text).	Thanks for this comment. We adjusted the last paragraph in the Methods section (p.8) to make it more clear why we think system dynamics is a valuable approach. We are convinced system dynamics is a powerful approach to study ED operations because (1) it adds to the diversity of analysis methods, (2) it is the natural analysis method when it comes to modeling patient flows and stock of patients (see explanations before), (3) due to its accessible user interface, the highly intuitive modeling iconography and the established methods in participatory modeling building (so-called scripts), SD might be the best approach when it comes to involve clients/stakeholders into the modeling process.
14	In Section 3.2 on the overall structure of the ED, some more information regarding the ED under study and the characteristics of the ED would be interesting, as there exists no general structure that represents every ED, and the effectiveness of improvement policies depends on ED characteristics (e.g. yearly number of patient arrivals, number of physicians working in each venue/shift, number of beds in the ED, percentages of patients per venue…). This might also enhance the understanding of the experiments and results presented in sections 4, 5 and 6.	Thanks for this comment. We included a new subsection on "ED under study" (see p. 8)

15	p8, first paragraph: “The higher the ED patient’s acuity or priority, the greater the average physician consultation time.”. How is this determined, based on real-life data, observations, literature?	This is based on real-life data from the ED under study in Singapore.
16	Figure 3: This figure also contains ‘treatment’ and ‘follow-up consultation’ as part of the patient flow through the ED. Are these also included in the simulation model? Based on the care pathways discussed in Section 3.3, all patients seem to have only one consultation with a physician, which does not represent the actual patient flow in most EDs.	Thanks for the comment. The follow-up of treatment and consultation is included in the average time used in the simulation model. In the simulation model, average time for consultation was used which includes patients that received follow-up for treatment. As a result, figure 3 is consistent with the simulation model presented in the manuscript.
17	The Model Structure section (Section 3.3) is difficult to read and understand because of the many equations and abbreviations. Depending on the main contribution of the paper (i.e. system dynamics or policy investigation), this section can be shortened and some equations can be placed in the appendix. For example, the four care pathways in the critical care area (Section 3.3.2) are all possible pathways in the ED (i.e. no new pathways are introduced in the other two zones). As the pathways and accompanying mathematical equations are discussed in great detail in Section 3.3.2, the description in Sections 3.3.3 and 3.3.5 can be shortened by indicating that the reasoning and equations are comparable to the pathways in the critical care area, but with other parameter values. In addition, the abbreviations make it difficult to understand the mathematical equations and model description. Would it be possible to provide an explanation of all the stocks and flows in words, in addition to the mathematical equations? Especially for non-experts (e.g. ED staff) this might be useful to better understand the model.	Thanks for the comment. Upon careful consideration of your suggestion, we have decided to maintain the equations in the main manuscript to prevent a disruption of the flow to potential readers.

18	p9, first paragraph: "… developed from a broad range of empirical data.". What empirical data is used in this study? As the appendix contains a table with all model parameters and their source, a reference to this table should be added to the text.	This is more a general statement that system dynamics models are parameterized from a broad range of empirical data.
19	p13, second paragraph: "… AT is adjustment time …". What is meant with adjustment time?	Thanks for the comment. Adjustment time is a model artifact to ensure unit consistency. The value is 1 and it does not have any impact on the equations used but to ensure unit consistency.
20	p14, equation (33): As POW refers to all patients under observation in the waiting area, shouldn't this equation only contain the fraction of patients that is discharged home, because POW also contains admitted patients and these are not discharged home? An explanation in words would enhance the understandability of the equations.	Thanks for the comment. POW includes patients discharged home as well as patients admitted. POW (patients under observation in waiting area) only occurs when the observation ward is full, thus it could include patients that could be discharged as well as admitted. In page 14 we have a sentence that makes clear what POW includes..."Patients under observation in the waiting area 〚POW〛_j (t) are discharged 〚dw〛_j (t) as their conditions improve or admitted to the hospital cad(t)".
21	Section 3.3.3: Why is 'AB' added to all abbreviations to refer to ambulatory care (and IS to refer to isolation care in Section 3.3.5), while no reference to critical care is added to the abbreviations in Section 3.3.2? Try to be consistent, as this will enhance the understanding of the many abbreviations.	Thanks for the comment. We have provided a detailed list of the abbreviations used in the manuscript in Table 5 in the Supporting Information. This we believe will address your question to ensure that there is no confusion as to the meaning of the abbreviations used. we are confident that the current presentation will not create any confusion for potential readers of the manuscript.
22	The Data sources section (Section 3.4) is very short, especially as very little information is provided within the other sections on the characteristics of the ED under study, the data used to determine model parameters and the values of these parameters.	Thanks for this comment. As previously mentioned, we included a new section "ED under study" into the manuscript to provide more specifics on the modeled ED (see p.8). Also, in the section on "Data sources" we added a sentence to refer the reader to the Supporting Information to get more information on model inputs, their values, units, and sources (see p. 24)
23	p22, last paragraph: "The patient arrival data used in the simulation model was the highest patient arrival pattern during the period of June – August 2017 …". Does this arrival pattern consist of just one day of patient arrivals? Or one month? What are the run parameters of the simulation model? And why only using the highest arrival pattern, is this representative for actual ED operations?	Thanks for this comment. The model runs for 24 hours. We had access to ED data from June - August 2017. We picked the highest arrival pattern in this 3-months period because we wanted to stress-test the system. Operational problems in the ED only appear when the workload is very high, and the system is stressed to its limits.

24	P23, last sentence of Section 3.4: “The simulation model is provided in the supplementary materials for review.”. There was no supplementary material available regarding the simulation model.	Thanks for this comment. We uploaded the model as a supplementary file. The simulation model is programmed within Vensim DSS.
25	In the Model validation section (Section 3.5), a more comprehensive explanation of the statistical tests is required to better understand the validation process. How to interpret MAPE, and how to determine whether the MAPE value indicates a good fit between the simulation model and actual data? The same for the Theil statistic, how to interpret the U-values and how to determine whether they represent a good fit?	Thanks for the comment. We have included explanation the MAPE to help the readers of the manuscript determine how good our estimated MAPE are. In addition, we have given further explanation for the Theil statistics.
26	p23, second paragraph of section 3.5: The behavior validation tests only look at the average length of stay across all venues. However, when the variability in LOS within and across venues (and time periods) is high, the average LOS of the simulation model and data can be comparable, while the simulation model poorly represents actual ED operations	Thanks for the comment. We used average length of stay across all venues because it was the outcome variable that was agreed upon by the ED doctors we engaged in this process. In addition, we feel this is an appropriate outcome variable notwithstanding the likely variability as indicated by the reviewer.
27	p23, last sentence: “This suggests that the simulated variables track the underlying trend well, but diverge when comparing point-by-point, indicating that majority of the errors are unsystematic with respect to the purpose of the model.”. Explain? And what does the difference in U-values between the critical care area, ambulatory care area and isolation care area mean? Does the model only provide a good fit for some of the areas?	Thanks for the comment. The sentence indicates that when considering Theil Statistics, it is important to look at all the errors presented i.e. the covariation component (UC), bias (UM) and unequal variance (US). However, the covariation error indicates unsystematic error. The results show that the model provides good fit for all the outcome variable i.e. average length of stay.
28	Regarding the Policy Experiments section, some information on the reason for choosing these policies is lacking. What is the current ED performance and what are the bottlenecks? How are the experiments determined in order to resolve these bottlenecks?	Thanks for the comments. We have included the rationale for the policies in the policy experiment section. We indicated that "Policy experimentation was conducted to explore the range of potential future directions on how to manage ED crowding as discussed with the ED physicians, as well as address the identified bottlenecks—significant waiting times for consultation, laboratory investigation and observation ward for admission—in the ED care processes".

29	Base case scenario: In the text, this scenario is referred to as a ‘hypothetical scenario’, but isn’t this the current ED setting?	Thanks for this comment. This is the "business-as-usual" scenario, i.e., stick to the status quo, which is rather unrealistic (and so hypothetical) because changes to the status quo can be expected in the coming years (due to new policies being implemented)
30	Policy 3: An increase by 20 percent seems a very high increase, given the fact that the number of patients in the ED decreases through co-location. Is an equal increase in the number of ED physicians required in all venues and at all times (e.g. the average LOS is higher in phase 2 than in the other phases)? In the results section, it appears that the introduction of additional physician capacity is not capable of reducing LOS for all patients in the ED.	We changed the description of this policy and the other policies as well (see p.26/27). The idea of this staffing policy is to check by how much manpower needs to be increased in order to reduce the ALOS below the 4-hour target (4.5 h target for isolation care patients) across the entire day.
31	The Results section contains a lot of interesting results, but an explanation of the results is lacking. What explains the large differences in LOS between different care pathways and time intervals? Why are certain policies more effective to reduce LOS? And why do some care pathways benefit more from a certain policy? In addition, are the improvements in LOS statistically significant?	Thanks for the comment. We explained the results in the discussion section.
32	Based on the results in Table 1, policy 1 seems least effective. Why do the authors only focus on this policy in the conclusion section?	Thanks for the comment. It is important to emphasize that some policies are effective for ED patients that go through specific pathways only. Thus, policy 1 is effective for venues of care that have P4 and P3 patients. We discussed why we believe only ambulatory care have P4 and P3 patients--i.e. due to up-coding.
33	Figure 10: The base case scenario seems lacking on these graphs.	Thanks for this comment. We produced new Figures illustrating the impact of the four policies.
34	Figure 10 and 11: How can the large fluctuations in LOS be explained (even within a time period/phase)?	Thanks for the comment. The large fluctuations are mainly due to arrival time and number of ED doctors at post during specific times of the day.
35	Figure 11, isolation pathways: The target time is not presented on the graph regarding isolation pathway 1, probably because of the scale on the y-axis. Why is the scale on the y-axis different on both graphs of the isolation pathways?	We produced new Figures (Figs 10-13) in the Supporting Information illustrating the impact on ALOS of the different policies. We adjusted the scale of the axis such that they are homogenous for each care venue.

36	- In the Discussion and Conclusions sections, the focus is on the insights regarding the co-location of general practitioners in the ED. I agree that this is a very interesting improvement option to reduce ED crowding, but are there no main insights regarding the other policies and the SD model? The conclusions should provide an overview of the main contributions of the paper, and based on the current conclusions the only contribution seems to be the investigation of the co-location of general practitioners in the ED. If this is the main contribution, the introduction and literature review section should be rewritten to focus on this contribution (e.g. what literature does already exist on the use of GPs in an ED, and what is the added value of this study compared to existing literature?). Furthermore, a critical reflection on how the investigated policies can be implemented in practice, and on the generalizability of the results, is lacking.	Thanks for this comment. We adapted the "General remarks" section (p.32) to provide a discussion around the implications of all policies tested.
37	p29, last paragraph: "In addition, raising the number of ED doctors (via the dynamic allocation policy) …". The investigated policy seems no dynamic allocation policy, as only a 20% increase in physician capacity for all venues and time periods is investigated.	Thank you. We changed this particular sentence (p. 33).
38	p30, first paragraph of Section 6.2: "The key finding that decanting non-emergency patients who seek care at the ED to a GP clinic co-located at the ED significantly decreases ALOS and improves patient experience has policy implications." Is this the key finding? Based on the results in Table 1, this policy seems least effective to reduce LOS.	Thank you for this comment. The policy of co-locating primary care services within the ED is indeed the most effective in our experiments. When you look at Figure 10 (and in particular at graphs "Ambulatory pathway 1" and "Ambulatory pathway 2") in the Supporting Information you see that if you just treat 10% of all ambulatory patients (P4 and P3 patients) within a adjacent primary care clinic the impact on ALOS of the remaining ambulatory patients is massive. Because ambulatory patients make up the biggest share of all patients visits this seems to be the most promising policy.

39	p30, first paragraph of Section 6.2: “The lesson from this finding is that, there is a significant proportion of ED patients who inappropriately self-refer to the ED.” Is this true? The policy investigates the effect of referring 50% of the P3 and P4 patients to a GP, but does 50% of these patients inappropriately go to the ED for care? How is this percentage determined?	Yes this is true. There is a significant number of patients that need only primary care services. In Singapore and in other developed countries like Switzerland, emergency departments are not built to provide primary care but to provide urgent critical care to severely ill patients. Due to changes in patient behavior (and expectation), access and reputation of primary care providers, and an increase in chronic patients among other factors, emergency departments have started to provide more and more primary care distorting their main mission of providing life saving care. In Singapore, all P4 patients and at least half of the P3 patients could be handled by GPs. This number has been mentioned in several interviews with emergency physicians and nurses.
40	Section 6.3: The advantages of using SD do not become clear from this section (e.g. SD results in an oversimplification of actual ED operations).	Thanks for this comment. As mentioned previously we have adapted the section on methods to emphasize better the advantages of SD in modeling ED operations
41	Section 7: A paragraph on future research opportunities is lacking.	This project is already a follow-up study building on a previous work on a qualitative model on ED crowding in Singapore. We feel that overall this project is completed. As already mentioned there is a similar project in Switzerland - based on the model in this article where we try to slightly adapt the model to the new local context and test policies that are relevant for Switzerland.
