# Supplementary material for: Modeling Emergency Department crowding: Restoring the balance between demand for and supply of emergency medicine
Source: PLoS One. 2021 Jan 12;16(1):e0244097. doi: 10.1371/journal.pone.0244097 (PMC7802975; doi:10.1371/journal.pone.0244097)
Supplement: Attachment — Submitted filename: Response to Reviewers_R2.pdf [file pone.0244097.s008.pdf]

Dear editor & dear reviewer,

We thank you for your comments. The comments were valid and constructive and helped to improve our paper. All changes with respect to the last version are marked in green color. Thanks again for your time and effort to evaluate our paper.

Comments from reviewer		
**Nr.**	**Comment**	**Response**
1	Section 2<br>- The contributions of the paper are clarified in the introduction, but the added value of the literature review section is still rather limited. As indicated by the authors, an extensive literature review is not the goal of the paper and also not required. However, it is indicated that 18 papers exist on the application of SD in an ED context, while only 6 papers are discussed. At least, an explanation should be provided for only discussing these 6 papers in detail: Why are these papers most relevant to be discussed? In addition, a short discussion on how the current paper relates to the existing (and discussed) literature should be included at the end of the section to position the paper within the state-of-the-art literature.	Thanks for this comment.<br><br>On p.6, first paragraph, we added a sentence why we chose these 7 studies among the 18 SD works detected in the systematic literature review. In our literature review, we focused on works that are published in renowned international operational research (OR) or emergency medicine journals. So, we excluded conference proceedings and articles that have been published in journals with no impact factor or outside OR and emergency medicine. While screening the systematic literature review, we noticed that one additional article seems relevant for our study. Hence, we included one more article and presented its main findings in our literature review (p.6/7).<br>Furthermore, as suggested by the reviewer, we added a new paragraph at the end of the literature review (p.7) in order to explicitly link the review to the purpose of our article.
2	- The term 'adjustment time' should be clarified within the paper. In the response to review comments (comment 19) an explanation is provided, but this explanation should also be included in the paper such that readers understand this term.	Thanks for this comment.<br><br>We added an explanation of adjustment time (AT) in the main text (p. 14).
3	- Section 3.5, p23: "The patient arrival data used in the simulation model was the highest patient arrival pattern during the period…". In the response to reviewer comments (comment 23), it became clear that a 24 hour arrival pattern corresponding to a peak day in patient arrivals was used as input to the simulation model, in order to clearly represent the operational problems in the ED. However, this is still not clear from the text, so it might be interesting to include the explanation of the response to reviewer comments in the text.	Thanks for this comment.<br><br>We added this information on p.23/24

4	- Section 3.5: Patient records and observations are indicated as the two data sources, but expert opinion also seems an important source of input data based on S1 Table 5 and is currently not mentioned in this section.	Thanks for this comment.<br><br>We added the importance of expert judgment as a source of data on p.24
5	- Section 3.7: This section clearly adds value to the paper, but it can be integrated with Section 3.5, since both sections are rather short and deal with the use of input data to determine simulation model parameters.	Thanks for this comment.<br><br>We integrated section 3.7 into section 3.5 (see p.23/24)
6	- Section 3.6: The explanation of the statistics makes this section more clear, but in my opinion an explanation for the difference in Theil statistic value between the different care areas is still lacking. Why is there a difference between the care areas? And since the majority of errors for the ambulatory care area does not come from the covariation component, is the simulation model a good representation of actual operations in the ambulatory care area?	Thanks for this important comment.<br><br>In this section, we made a mistake. We forgot to recalculate MAPE and the Theil statistic after the first round of revision. We did this now and both measures changed significantly. This is the case because we exchanged single values (averages) for diverse process times with distributions in the first round of revision (see p.23/24). Now, both MAPE and the Theil statistic look better indicating a better fit with real operations in the ED. For the Theil statistic, now for all three venues of care the majority of the error can be attributed to the covariation component.
7	Section 4<br>- p26, policy 3: "This policy experiment gradually increases the number of ED doctors currently allocated to CCA, ambulatory care and isolation care from 10% to 30%..." This sentence is misleading in two different ways: (1) Based on this sentence, it seems that the number of allocated doctors is currently at 10% and gradually increases to 30%, but the base case scenario is a 0% increase; (2) In the next paragraphs, it becomes clear that the capacity of doctors is gradually increased by 10%, 20% and 30%, but based on this sentence it seems that the number of allocated doctors varies between 10% and 30%, but then the reader might pose the question: 10% or 30% of which doctors is allocated to the ED? Of the total amount of doctors in the hospital?. In order to avoid confusion, 'Optimal capacity of doctors' might be a better name for the policy, since the capacity of doctors is increased (no change in allocation of doctors between hospital departments).	Thanks for this comment.<br><br>We changed the respective sentence on p.26 to be more clear on the intention of policy 2. Also we changed the header of this policy to "Capacity of doctors".

8	o Section 5.2: The results for the ambulatory care area are only discussed for time phases 2 and 3, not for time phase 1 (00:00am- 08:00am).	Thanks for this comment.<br><br>Under policy 1,the simulation results revealed no change in ALOS in phase 1 (00:00 am – 08:00 am) for both ambulatory care pathways and across all scenarios. On p.28, we extended the description of results in order to be more detailed and comprehensive.
9	Section 5.5: Only 08:00am – 04:00pm is discussed for critical care pathway 1 under the 30% scenario,<br>00:00am-08:00am is lacking for ambulatory care pathway 1 under the 10% scenario.	Thanks for this comment.<br><br>We expanded our description of results in order that all time phases are discussed (see p.30/31)
10	- A lot of results are presented in this section, but an explanation of the results is sometimes lacking. An explanation can enhance the understanding of the results and support the main insights from the experiments.<br>o Section 5.2: Why is there no impact of policy 1 on isolation care pathways, since there are also P3 and P4 patients in this care area?	Thanks for this comment.<br><br>In general, we tried to provide more explanations in order to better understand the findings.<br><br>In the text, on p. 28, we make the following point: "Lastly, policy 1 does not show an impact on isolation care pathways in our experimental set-up. On average, only 6% of all presenting patients are triaged to isolation care (i.e. fever patients) and so patient flow is smooth. This means that fever patients typically do not need to wait until they see a doctor. Consequently, reducing the inflow of patients into isolation care (which is the effect of policy 1) does not change the ALOS of patients going through this venue of care. ALOS is driven by the waiting time for lab and investigation and if necessary, by the waiting time for a bed in the hospital. Both waiting times are not influenced by policy 1."
11	o Section 5.3: Why is there no influence on the ALOS of isolation care patients? The capacity of physicians also increased in this area?	Thanks for this comment.<br><br>We added an explanation on p. 29. The explanation is similar to the one given above. We wrote that "The drivers of ALOS for patients in isolation care are the waiting times for lab and investigation and admission to the hospital. Both waiting times are not changed (reduced) by increasing the capacity of doctors in the ED."
12	o Section 5.4: There seems to be only an influence on the ALOS for care pathways that include observation, not for care pathways that include laboratory examinations without observation. How can this be explained, as the waiting time for laboratory examinations is also reduced?	Thanks for this important comment.<br><br>Your comment is absolutely true. This was a mistake from our side because we left the time for "lab and investigation" constant throughout the scenarios – this was wrong. So we corrected this mistake and reduced the time for "lab and investigation" accordingly (reductions of 10%, 20%, and 30%). Hence, we reran the simulations and modified the text incl. Table 3 (section 5.4) and the Figures in the Supporting Information. Also we needed to reran experiments referring to policy 4

		because this mistake also had an impact on policy 4 simulations runs.
13	- Are the results regarding the comparison of the different policies with the BAU scenario statistically significant? In simulation there is a lot of randomness, which can for example be seen in the graphs of S1 Fig 10-13, so is it possible to compare the results of different policies only based on a mean value without statistical tests?	We think this is possible. A comparison of the means of ALOS is reasonable. In general, System Dynamics modeling is not such much concerned about maximal precision of predicted values but rather strives for predicted trends/modes of behavior of key (performance) parameters. Of course, simulation results could be further improved by Monte Carlo Simulation in order to derive confidence intervals around the modeling results.<br><br>We include this concern as a further limitation in the section on “Limitations of this study” (p. 35)
14	Section 6<br>- Section 6.2: From the text, it is still not clear why the insights regarding the co-location policy are the key findings of the paper. Other policies also seem efficient for several types of patients, sometimes even more efficient as they result in a higher reduction in ALOS. In the response to reviewer comments (comment 38), it is indicated that ambulatory care patients with priority P3 and P4 are the large majority of patients in the ED, and a small reduction in the number of patients from this category already highly impacts ED performance. This explanation should be included in this section to explain why this is indicated as the key finding of the study.	Thanks for this comment.<br><br>We included this reply/explanation on key finding in the main text (see p.34)
